# Reconstructing clonal tree for phylo-phenotypic characterization of cancer using single-cell transcriptomics

**DOI:** 10.1038/s41467-023-36202-y

**Published:** 2023-02-22

**Authors:** Seong-Hwan Jun, Hosein Toosi, Jeff Mold, Camilla Engblom, Xinsong Chen, Ciara O’Flanagan, Michael Hagemann-Jensen, Rickard Sandberg, Samuel Aparicio, Johan Hartman, Andrew Roth, Jens Lagergren

**Affiliations:** 1 SciLifeLab, School of EECS, KTH Royal Institute of Technology, Stockholm, Sweden; 2 Department of Cell and Molecular Biology, Karolinska Institutet, Solna, Sweden; 3 Department of Oncology and Pathology, Karolinska Institutet, Solna, Sweden; 4 Department of Molecular Oncology, BC Cancer, Vancouver, BC Canada; 5 Department of Pathology and Laboratory Medicine, University of British Columbia, Vancouver, Canada; 6 Department of Clinical Pathology and Cytology, Karolinska University Laboratory, Stockholm, Sweden; 7 Department of Computer Science, University of British Columbia, Vancouver, Canada; 8 Present Address: Department of Biostatistics and Computational Biology, University of Rochester Medical Center, Rochester, USA

**Keywords:** Phylogeny, Statistical methods, Cancer genomics, Data integration, Gynaecological cancer

## Abstract

Functional characterization of the cancer clones can shed light on the evolutionary mechanisms driving cancer’s proliferation and relapse mechanisms. Single-cell RNA sequencing data provide grounds for understanding the functional state of cancer as a whole; however, much research remains to identify and reconstruct clonal relationships toward characterizing the changes in functions of individual clones. We present PhylEx that integrates bulk genomics data with co-occurrences of mutations from single-cell RNA sequencing data to reconstruct high-fidelity clonal trees. We evaluate PhylEx on synthetic and well-characterized high-grade serous ovarian cancer cell line datasets. PhylEx outperforms the state-of-the-art methods both when comparing capacity for clonal tree reconstruction and for identifying clones. We analyze high-grade serous ovarian cancer and breast cancer data to show that PhylEx exploits clonal expression profiles beyond what is possible with expression-based clustering methods and clear the way for accurate inference of clonal trees and robust phylo-phenotypic analysis of cancer.

## Abstract (web summary)

The functional changes of individual clones in single cell RNA sequencing (scRNA-seq) data remain elusive. Here, the authors develop PhylEx that integrates bulk genomics data with co-occurrences of mutations revealed by scRNA-seq data and apply it to high-grade serous ovarian cancer cell line and breast cancer datasets.

## Introduction

Cancer is an evolutionary process with ongoing mutational processes coupled with selection and drift leading to genetic diversity within the tumor cell populations. Though each cell is fundamentally distinct in cancer, there typically exist groups of cells that are genomically nearly identical, so-called clonal populations^1^. The evolutionary relationship between clones can be represented by a phylogenetic tree or clonal tree. Inferring clonal population structure, genotypes, and trees from sequence data has been an active area of research in the past decade with implications for cancer treatment^2–4^. Early approaches used bulk sequence data coupled with computational deconvolution to address the admixed nature of bulk data^5–9^. The limitations of clonal analysis using only the bulk method is well documented in the literature (e.g., refs. ^10,11^). Recent advances in single-cell DNA sequencing (scDNA-seq) technologies have prompted the development of approaches better tailored to these data types^12–14^ as well as methods that integrate scDNA-seq data with the bulk data for joint analysis for improved accuracy^15,16^.

Though the aforementioned methods can resolve clonal population structure, they cannot identify functional differences that result from the genomic heterogeneity. The increasing availability of single-cell RNA sequencing (scRNA-seq) data provides an approach to partially address this problem. Recent methods that seek to assign gene expression profiles to clones have treated the problem as a two-step procedure whereby the clonal population structure is identified and then scRNA data is aligned to clonal genotypes^17,18^. However, the two-step approach does not fully utilize the available data as information in the scRNA data cannot be used to improve clonal population structure. Hence, there is an unmet need for an integrative approach to simultaneously identify clonal population structure and the associated clonal genotypes from bulk DNA- and scRNA-seq data towards identifying intra-tumor heterogeneity in clonal gene expression profiles.

In this work, we introduce a Bayesian probabilistic method called PhylEx (**Phyl**o **Ex**pression) that integrates bulk DNA- and scRNA-seq data to meet this need. PhylEx leverages information about the single-nucleotide variants (SNVs) observed within a single cell to identify clones, improve clonal tree reconstruction, and facilitate highly accurate mapping of RNA expression profiles to clones. Thus, PhylEx unlocks the potential for *phylo-phenotypic* analysis, to discover and characterize tumor’s progression and relapse mechanisms at gene and functional (pathway) levels of individual clones in relation to clonal genotypes, within an evolutionary context. We systematically benchmark PhylEx using synthetic data and compare it to existing state-of-the-art clone reconstruction methods. We then evaluate the performance of PhylEx on high-grade serous ovarian cancer (HGSOC) cell lines, which were thoroughly investigated using the direct library preparation (DLP) scDNA approach in ref. ^19^. The experimental results demonstrate that integration of bulk DNA and scRNA allows for the identification of clonal population structure with high fidelity. Finally, we apply PhylEx to breast cancer data along with HGSOC cell line to characterize patterns of cancer progression using the clonal expression profiles.

## Results

### Method overview

**Fig. 1:**
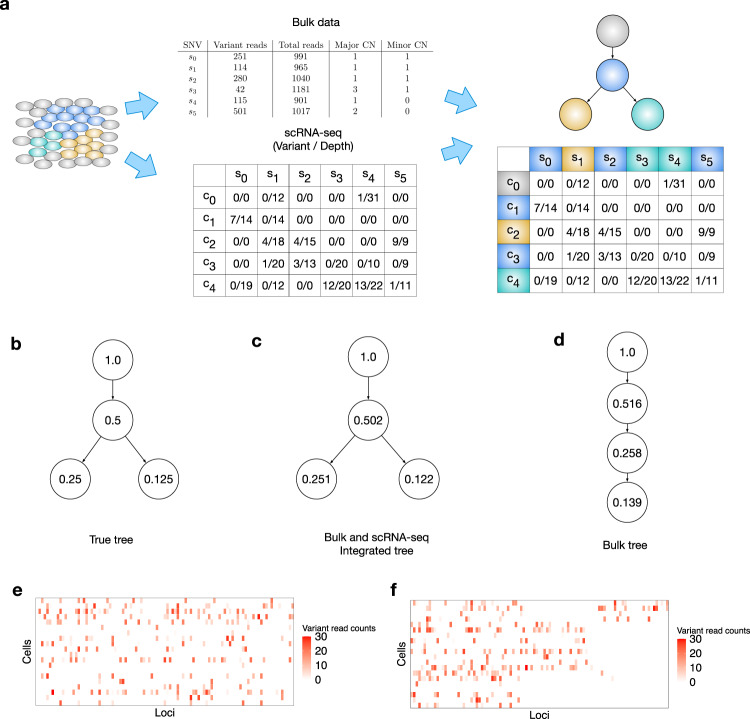
Overview of PhylEx. **a** Schematic diagram describing the bulk DNA-seq and scRNA-seq data input. The output of PhylEx includes the tree and assignment of SNVs and cells to clones. **b** The cherry shaped tree used in the illustrative example for identifying branching structure from scRNA-seq. The true values of the cellular prevalences are indicated for each clone. **c** Inferred tree and cellular prevalences from integrated analysis of bulk DNA-seq and scRNA-seq. **d** Inferred tree and cellular prevalences using bulk DNA-seq. **e** The heatmap of the variant read counts of single-cells across loci and **f**. the heatmap of the variant read counts of single cells after co-clustering of cells and SNVs using PhylEx. All the cells share common set of ancestral SNVs and we can see two clusters of cells based on their clonal membership. Source data for **e**, **f** are provided as a Source Data file.

PhylEx is a Bayesian statistical method that simultaneously reconstructs a clonal tree and assigns single-cells, as well as genotypes, to the clones for a tumor characterized by bulk DNA-seq and scRNA-seq data (Fig. 1a). Standard bulk data processing is performed, including variant and copy number calls to identify loci with SNVs and their copy number profiles. The bulk data consists of the number of reads mapping to the variant allele and the total number of reads mapping to each locus. Similarly, standard scRNA-seq data processing is applied to align and map the reads for each cell, yielding data that consists of the total depth and the number of reads mapping to the variant allele for each locus (“Methods” section).

The underlying statistical model is based on the tree-structured stick breaking (TSSB) process, a flexible prior distribution over the clonal tree structure^20^, and an infinite site model that define a distribution over clonal genotypes. The model has an observational component for the read counts from bulk DNA-seq and scRNA-seq data, conditional on the clonal tree and the genotypes associated with the clones. The observed variant read counts from the bulk data follow a binomial distribution parameterized by the read depth and probability of success parameterized by an unobserved cellular prevalence and an estimated clonal copy-number. The observed variant read counts from the scRNA-seq data are modeled using a mixture of two Beta-Binomial distributions, one for the mono-allelic and one for the bi-allelic expression. To sufficiently model the stochasticity in the scRNA-seq data, we have classified the biological processes that underlie scRNA-seq data into five categories: (1) zero expression (2) mono-allelic expression of the reference allele; (3) mono-allelic expression of the variant allele; (4) bi-allelic expression; and (5) no mutation and provided examples of each of the five categories in Supplementary Fig. 7h. The zero expression arises when no read maps to a locus. The scRNA-seq data is generally sparse and we expect a large number of zero expression (see for example, ref. ^21^, for a recent discussion on zeros in scRNA-seq data). The mono-allelic expression may arise due to bursty expression^22^, where a cell may express only the reference allele or the variant allele but not both. The bi-allelic expression arises when both alleles are expressed. Finally, expression of variant allele depends on the clonality of the cell. If a cell does not harbor a mutation at a locus, then a variant will only be observed in error be it at the stage of sequencing or bioinformatics processing. Our exploratory analyses of the real sequencing data shown in Supplementary Fig. 7a–g demonstrate that the scRNA-seq read counts arising from the aforementioned stochastic processes can be sufficiently modeled by a mixture Beta-Binomial distributions.

The inference machinery takes advantage of slice sampling to explore the space of clonal trees^23^ and Metropolis-Hastings for exploring the clone fractions^6,7^. PhylEx marginalizes over all possible cell-to-clone assignments to evaluate the likelihood of the single-cell data as marginalization has the positive effect of removing uncertainty in scoring the clonal tree due to latent cell-to-clone membership variables. PhylEx generates samples from the posterior distribution over the clonal tree as well as a *maximum a posteriori* (MAP) tree. The output also includes clonal genotypes and cell-to-clone assignments. The clone analysis conducted by PhylEx then facilitates a range of differential expression investigations on the otherwise inaccessible tumor clones.

### Related methods

There have been several approaches that have considered integrating single-cell and bulk sequencing data. The method most closely related to PhylEx is ddClone, which performs Bayesian inference of clonal structure using an integrated likelihood for bulk and single-cell data^15^. In contrast to our method, ddClone uses scDNA-seq data and as such, ddClone cannot infer clonal gene expression profiles; furthermore, ddClone does not infer a clonal tree. B-SCITE also performs integrated analysis of bulk DNA- and scDNA-seq data but it targets mutation trees^16^; a post-processing step needs to be applied to convert mutation trees to (sub)clonal trees. As the two methods are tailored to work with scDNA-seq, they are not well suited to handle the stochasticity in scRNA-seq data, necessitating the development of a specialized method tailored for scRNA-seq. Namely, PhylEx is better equipped to account for sparse nature of the scRNA-seq data due to both biological and technical reasons and elevated false negative rates due to mono-allelic expression (Supplementary Fig. 7h).

Also closely related to PhylEx are approaches that consider the problem of mapping scRNA-seq data to clones. The earliest approach we are aware of is clonealign, which maps the gene expression profiles in scRNA-seq data to clonal copy number profiles^17^. In the original publication, the copy number profiles were inferred from scDNA data, though in principle they could also be inferred from bulk sequencing data. In contrast to PhylEx, clonealign does not infer a phylogeny as it assumes clonal tree along with clonal copy number profiles as given and fixed throughout inference procedure. Furthermore, clonealign requires that there is sufficient copy number variability between clones to uniquely correlate scRNA expression to genotypes. Thus clonealign is not applicable to cancers without significant copy number variation. Cardelino is another method for mapping scRNA-seq to clones; like PhylEx, Cardelino maps scRNA data to clones using SNVs^18^. Because both PhylEx and Cardelino uses the SNVs to facilitate mapping of scRNA-seq data to clones, the two methods can easily be mistaken as competitors. The primary goal of Cardelino is to infer the mapping of cells to clones and a clonal configuration matrix (i.e., assignment of SNVs to clones), given an initial clonal configuration matrix obtained from bulk DNA-seq data. Specifically, Cardelino represents clonal configuration by a binary matrix *C* ∈ {0, 1}^*N*×*K*^, where *N* denotes the number of SNVs, *K* denotes the number of clones and the entries *c*_*nk*_ ∈ {0, 1} indicates presence of mutation *n* = 1, ..., *N* in clone *k* = 1, ..., *K*. Cardelino gains some flexibility from a poorly constructed initial clonal configuration matrix by re-assigning SNVS to clones; nonetheless, the mapping of cells to clones is sensitive to the initial clonal configuration matrix, which is noisy if inferred purely from bulk DNA-seq data as we demonstrate in this paper. In contrast, the primary goal of PhylEx is to infer a clonal tree to reveal an evolutionary process underlying cancer progression. As the clonal tree yields clonal configuration matrix, PhylEx also provides high fidelity clonal configuration matrix to facilitate the mapping of cells to the clones. Note that workflow presented by Cardelino represents a two-step approach to mapping scRNA-seq data: first step involves inferring the clonal structure using bulk DNA-seq, followed by the second step of mapping scRNA-seq data to the discovered clones. We show that inaccuracies from the first step propagate to the second step, resulting in an inaccurate mapping (Supplementary Fig. 2) and that PhylEx alleviates this inefficiency by integrating bulk DNA and scRNA data likelihoods to infer clonal trees.

Finally, some methods perform *de novo* reconstruction of clonal configuration matrix or single-cell phylogeny from scRNA-seq data alone. Two methods that we are aware of are Cardelino-free and DENDRO^18,24^. Carlino-free appears to function similarly to Cardelino (a detailed method description is missing in the original publication): for a given number of clones, *K*, Cardelino-free reconstructs the clonal configuration matrix by assigning each of *N* SNVs and single cells to to one of the *K* pre-specified clones. This procedure does not involve bulk data likelihood nor an initial clonal configuration matrix inferred from bulk DNA-seq data. The authors only recommend using Cardelino-free when bulk data is missing as the performance of Cardelino-free is found to be inferior to Cardelino (p. 416 of ref. ^18^). It is important to note that (i) Cardelino-free serves a different use case than that of PhylEx, and (ii) the target of inference is a clonal configuration matrix as opposed to a clonal tree. DENDRO also differs from PhylEx in two ways. First, it reconstructs a single cell phylogeny. Second, it uses only scRNA-seq data (i.e., it does not involve an integrative data likelihood for bulk DNA and scRNA data). De novo reconstruction of single-cell phylogeny from scRNA-seq data is inherently challenging due to high levels of sparsity and missingness. By directly targetting clonal trees and integrating bulk DNA-seq data likelihood, PhylEx aims to alleviate these concerns.

### Integrating scRNA with bulk DNA data improves clonal tree reconstruction

We begin with an illustrative example to test the strength of the co-occurrence signal in single-cell data. We simulated bulk and scRNA-seq data for 100 SNVs and 20 single-cells over a cherry shaped tree (Fig. 1b) under an evolutionary model devoid of copy-number aberrations. We analyzed this data using PhylEx and compared it to bulk-based clonal tree reconstruction method PhyloWGS^7^. Figure 1c, d show the MAP trees from PhylEx and PhyloWGS respectively. Both methods infer the cellular prevalences correctly, but the tree inferred by PhyloWGS is linear as the observed bulk variant allele frequencies (VAFs) are equally well explained by the linear tree. In contrast, PhylEx correctly infers the cherry shaped tree by taking advantage of the co-occurrence of mutations in the single-cell data and performs co-clustering of the SNVs and cells (Fig. 1e, f). This example highlights that estimating clonal trees from bulk DNA data alone is an unidentifiable problem.

**Fig. 2:**
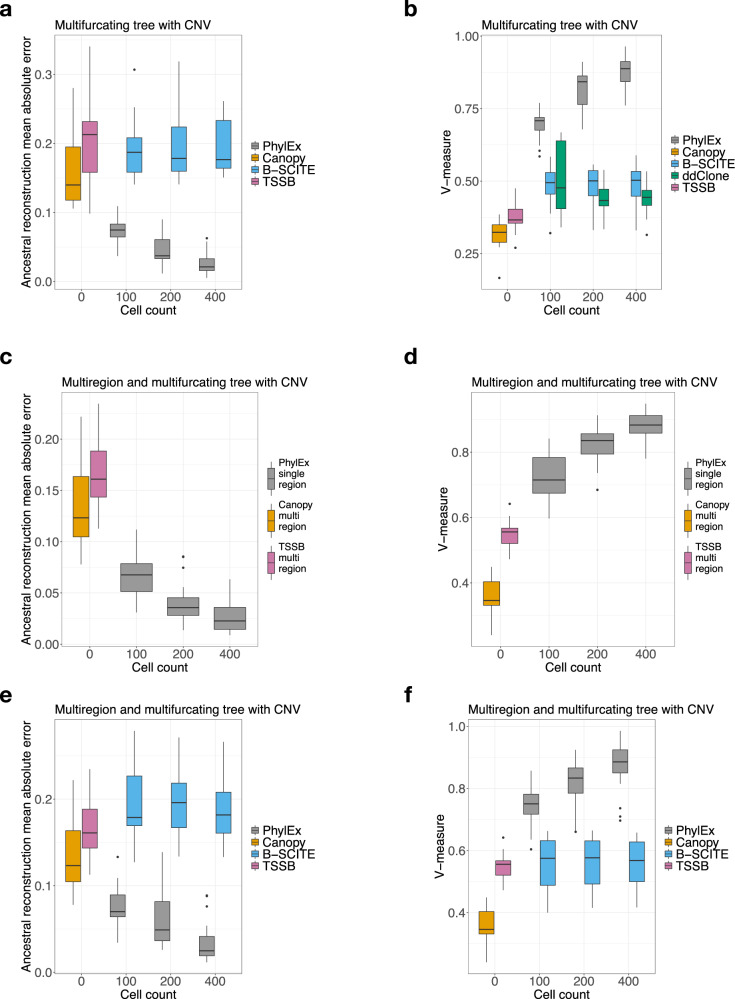
Simulated data analysis results with 20 data replicates generated with 100 SNVs in each replicate from multifurcating tree with copy number evolution. **a**, **b** Compared PhylEx to competitors on tree reconstruction error and on V-measure. **c**, **d** Comparison of PhylEx using single-region bulk DNA-seq and scRNA-seq to bulk-based methods supplied with multi-region DNA-seq using tree reconstruction error and V-measure. **e**, **f** Comparison of PhylEx on multi-region bulk DNA and scRNA data to the competitors. The box plot shows the median and inter-quantile range (IQR) at the 1st and the 3rd quantiles; the top (bottom) whisker indicates the maximal (minimal) point no further than 1.5 × IQR from the third (first) quantile. Source data are provided as a Source Data file.

We performed a comprehensive study of simulated data on larger trees and a model of evolution involving copy-number changes. As the cancer evolution can involve multifurcating events^25^, we simulated the data using multifurcating trees as well as a binary tree (Supplementary Section 2.1). Recall that copy-number variation obfuscate the VAFs, which renders bulk data-based clonal tree reconstruction an underdetermined problem. We compared PhylEx to clonal tree reconstruction methods PhyloWGS and Canopy. PhyloWGS requires subclonal copy number calls as an input; since such data is not available for simulated data, we implemented the methodology underlying PhyloWGS, which we refer to as TSSB, to investigate the performance of the PhyloWGS methodology. Canopy^9^ is a Bayesian clonal tree reconstruction software that takes advantage of clonal copy-number information. Canopy has previously been used for single-cell gene expression analyses that require a clonal tree as input (e.g., Cardelino^18^). We used V-measure and the ancestral reconstruction error given in Eq. (14) as evaluation metrics. We found that for both binary and multifurcating trees, PhylEx outperformed Canopy and PhyloWGS/TSSB (Fig. 2a, b and Supplementary Fig. 1a, b)). The performance of PhylEx improves progressively with the number of cells, as hoped. Comparing PhylEx to bulk-based clonal tree reconstruction methods further demonstrates that scRNA-seq data can mitigate the impact of (subclonal) copy-number changes on clonal tree reconstruction accuracy.

We conducted additional analyses to illustrate the potential pitfall of mapping scRNA-seq data to clones using a two stage approach. We follow the two stage approach proposed in ref. ^18^ where a clonal tree is inferred using Canopy using bulk sequencing data alone, and used as an input to Cardelino, which we refer to as CanopyCardelino. We inferred a clonal tree using PhylEx and inputted the inferred clones to Cardelino and PhylEx’s own mapping algorithm, respectively referred to as PhylExCardelino and PhylEx. Recall that mapping of cells to clones admits calling the genotypes of each cell (i.e., the presence and absence of SNVs in each individual cell). Hence, we compared expected loss, which roughly translates as an average number of SNVs incorrectly predicted for each cell (defined in Eq. (16)). The experiment was performed on simulated data from binary trees with and without copy number evolution (Supplementary Fig. 2). The results are poor for the two stage method – that even a sophisticated mapping algorithm such as Cardelino cannot overcome poorly constructed clonal tree. However, given a high fidelity clonal tree, PhylEx’s mapping algorithm does well and more importantly, the performance improves as more cells are added. Also note from the results that Cardelino achieves the state-of-the-art performance – this is unsurprising as mentioned in the Related methods section, that Cardelino refines clonal genotype configuration matrix to achieve the best possible mapping of scRNA-seq data to clones. In contrast, PhylEx’s mapping algorithm does not refine clonal configuration matrix. Our recommendation is to use PhylEx to infer the clonal trees and hence, the clonal configuration matrix coupled with Cardelino to map cells to clones.

### PhylEx reconstructs high fidelity clonal trees from single-region bulk DNA-seq integrated with scRNA-seq evaluated on synthetic data

Multi-region sequencing is a standard approach to improve the accuracy of the clonal tree reconstruction, e.g., to resolve branching^3,5–7,26^. For solid tumors, spatial samples are taken as statistical replicates with common evolutionary history but possibly with different cellular prevalences. However, depending on the type of tumor, spatial sampling may not be feasible. In particular, multi-regional sampling is difficult to perform without prior surgical tumor removal, preventing it from impacting pre-surgical treatment decisions. We evaluated the performance of PhylEx on simulated data consisting of a single-region bulk DNA-seq combined with scRNA-seq data against bulk methods supplied with multi-region DNA data.

We used a multifurcating tree and simulated the bulk DNA data with and without copy number variation. Devoid of copy number evolution and given multi-region data, the bulk methods achieved high accuracy (Supplementary Fig. 1c, d): for example, PhyloWGS and TSSB achieved 0.85 in the V-measure metric on multifurcating trees. Nevertheless, when supplied with single-cell data, PhylEx performed better, achieving a V-measure metric upwards of 0.95 using 400 cells (Supplementary Fig. 1e, f). With data simulated under a copy-number evolution model, bulk clonal tree reconstruction methods struggled even when supplied with multi-region data. On the contrary, PhylEx improved the accuracy given only a single-region bulk DNA-seq integrated with scRNA-seq data in the analysis (Fig. 2c, d). This investigation shows that PhylEx reconstructs high-quality clonal trees using single region bulk DNA and scRNA sequencing, increasing applicability of clonal tree reconstruction methods to various research and clinical settings that are limited to single-region sequencing.

### Investigation on synthetic data reveals that a specialized method to integrate bulk and scRNA-seq is necessary to overcome the limitations of existing bulk and scDNA-seq integration methods

Next, we compared PhylEx to two methods that integrate bulk genomics data with scDNA-seq data, B-SCITE and ddClone^15,16^, on synthetic data. One of the challenges of using these methods is that they require cell genotyping as a pre-processing step, i.e., to determine the presence or absence of mutation for each cell at each of the identified SNV loci. Although cell genotyping is an active field of research, it remains a challenging problem with the potential for high false positive (FP) and false-negative (FN) rates, especially when applied to scRNA-seq data as the expression profile is inherently sparse, bursty, with frequent mono-allelic expression. One of the key features of PhylEx is that it works with the raw read counts and does not require cell genotyping.

We found that PhylEx outperformed ddClone and B-SCITE on synthetic data generated from both binary and multifurcating trees, under evolutionary models with and without copy numbers aberrations (Fig. 2a, b, e, f and Supplementary Fig. 1a, b, e, f). Importantly, PhylEx exhibited an increase in performance with an increasing number of cells. In contrast, the other methods did not benefit from having more cells, likely because having more cells implies a higher incidence of FP and FN variant calls. Our results suggest that specialized methods for integrating bulk genomics with single-cell transcriptomics are needed to extract the signal from scRNA-seq data and that given data for sufficiently many cells, PhylEx reconstructs the correct clonal tree.

### PhylEx reconstructs the lineage of high-grade serous ovarian cancer clones

To assess the performance of PhylEx on real data, we analyzed a related set of high-grade serous ovarian cancer cell-lines^27^. The cell-lines are derived from the same patient, one from the primary tumor (OV2295) and two from relapse specimens (OV2295R2 and TOV2295R). These cell-lines have been assayed using the direct library preparation (DLP) scDNA-seq technology^28^ and analyzed by some of the leading experts in the field of computational oncology using multiple genomic characteristics, e.g., copy-numbers, breakpoints, and SNVs, to reconstruct a clonal tree, which we call DLP clonal tree (Fig. 3h in ref. ^19^). Since their evidence-based analysis was supported by multiple genomic characteristics, DLP clonal tree must be considered very solid, providing a fertile opportunity to assess performance of PhylEx on real sequencing data. To that end, we performed Smart-Seq3 scRNA-seq^29^ on OV2295 and OV2295R2 (TOV2295R was difficult to grow and we could not use it).

**Fig. 3:**
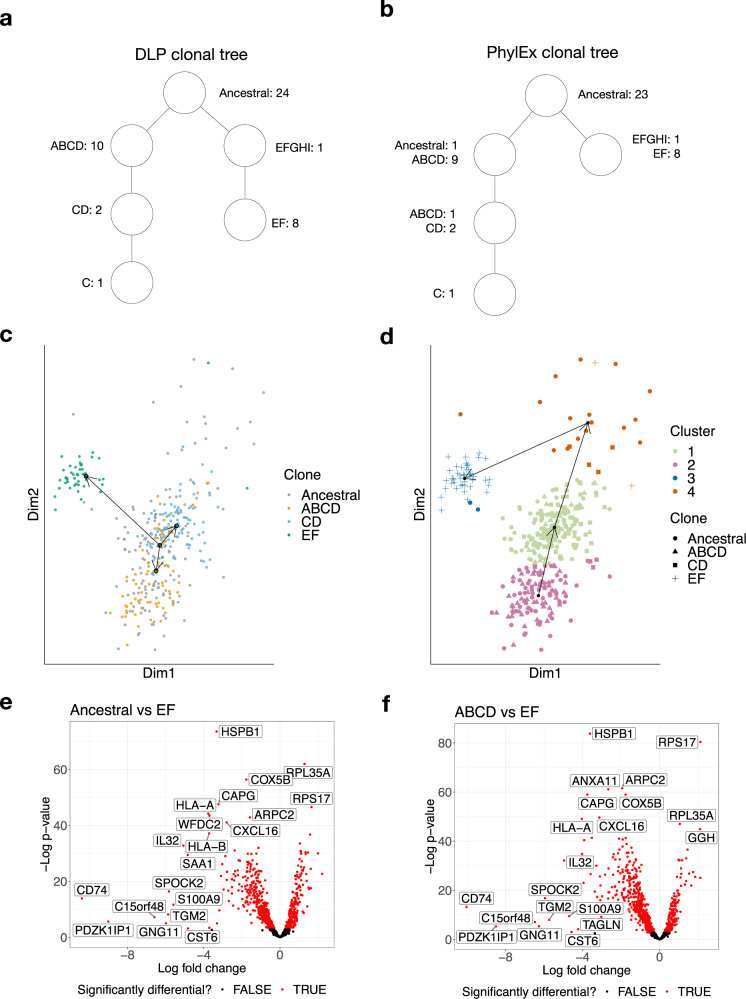
Analysis of HGSOC cell line. **a** DLP clonal tree with the number of SNVs assigned to each clone indicated beside the clone name. **b** The inferred tree from PhylEx; the number of SNVs attached to each node is obtained from the DLP clonal tree annotation in **a**. The plot of the gene expressions for cells on ZINB-WaVE dimensions: **c**. cells are color-coded after assigning to the clonal tree output from PhylEx, and the trajectory analysis result is overlaid on the figure with the ancestral clone specified as the starting cluster; **d** clustering of cells using mclust with the trajectory analysis with starting cluster unspecified. The visualization of differential gene expression analysis using volcano plots: **e** the EF clone to the Ancestral clone, and **f** the EF clone to the ABCD clone. Source data for a is provided as Supplementary Data 1. Source data for **b**–**f** are provided as a Source Data file.

We constructed a single-region pseudo-bulk data by combining the scDNA-seq data from the two cell-lines OV2295 and OV2295R2. We obtained 360 scRNA-seq cells passing quality control and identified 67 SNVs with coverage in the scRNA data. Of the 67 SNVs, 21 SNVs were removed from evaluation, but not the PhylEx analysis, due to incompatible annotation in the original publication^19^. To elaborate, an SNV is removed from evaluation only based on whether the authors of the original publication made consistent annotation with the tree that they inferred (Fig. 3h of ref. ^19^); inferred PhylEx tree was not used in determining which SNVs to remove. The annotation for each of the SNVs from ref. ^19^ is given in Supplementary Data 1 along with indication of which SNVs are excluded from evaluation. Alternative was to manually correct the inconsistent annotations and use all 67 SNVs in the evaluation; however, we deemed this process would be subject to bias.

There is a strong concordance between DLP clonal tree and the PhylEx MAP clonal tree. First, when disregarding a node of DLP clonal tree with a single SNV (labeled *EFGHI* in Fig. 3a), the trees have the identical topology (Fig. 3a, b). PhylEx correctly assigned 23 of 24 ancestral mutations. One SNV in *ABCD* clone was assigned to the *CD* clone. The clones *EF* and *EFGHI* were clumped together, thereby also clustering the lone SNV in *EFGHI* clone with the SNVs in *EF* clone. We compared the results of PhylEx to those inferred with Canopy, TSSB, ddClone, and B-SCITE^6,7,9,15,16^ on three clustering metrics and ancestral reconstruction metric. PhylEx significantly outperformed all of the other methods (Table 1). We have repeated the experiment under different parameter settings for PhylEx to demonstrate the robustness of the conclusion (Supplementary Table 3).

**Table 1:** Performance metric comparing PhylEx to Canopy, TSSB, B-SCITE, and ddClone on HGSOC data supplied with Smart-Seq3 scRNA-seq

Method	V-Measure	Adj. Rand Index	Adj. Mut Info	Anc. Recon Err
Canopy	0.494	0.386	0.327	0.178
ddClone	0.571	0.240	0.254	NA
B-SCITE	0.445	0.108	0.238	0.259
TSSB	0.237 ± 0.068	0.283 ± 0.077	0.180 ± 0.075	0.204 ± 0.032
PhylEx	**0.870** ± **0.0132**	**0.888** ± **0.0164**	**0.839** ± **0.0175**	**0.0379** ± **0.0077**

Used 20 runs for PhylEx and TSSB. Canopy and B-SCITE were ran with four MCMC chains. The first column lists the name of the methods. The second to fourth columns are clustering metrics used for comparison. The last column is the ancestral reconstruction error metric. The boldface indicates the best performing method. Source data are provided as a Source Data file.

To demonstrate a potential problem of two-step approach, we applied Cardelino to assign cells to the clones inferred from Canopy. The mutations that Canopy identified as exclusive to Clones 6 and 8 are marked in Supplementary Fig. 4e. However, we found cells assigned to other clones frequently carried mutations on these loci (Supplementary Fig. 3a). This is in a stark contrast to cells assigned by PhylEx (Supplementary Fig. 3b) with clear partition of cells by clones and their genotypes.

### Phylo-phenotypic analysis reveals immunoediting in metastases

To demonstrate PhylEx’s ability to perform phylo-phenotypic analysis, we performed gene expression analysis on clones discovered by PhylEx on the HGSOC Smart-Seq3 scRNA-seq data. We cannot evaluate the correctness of cell-to-clone assignment as ground truth does not exist. However, the co-clustering of SNVs and the cells to clones indicates its correctness (Supplementary Fig. 3b). Namely, we observed that all cells shared the ancestral SNVs (Ancestral clone) while the cells assigned to the clone in the relapse tumor did not express the SNVs in the primary tumor and vice versa.

We selected 1000 genes with the most variable expression pattern for differential gene analysis. We used a zero-inflated negative binomial model (ZINB-WaVE)^30^ to reduce the dimensionality of the gene expressions data to 2-dimensions. There was a clear separation between the expression of the EF clade (OV2295R) and the primary ABCD clade (OV2295) (Fig. 3c and Supplementary Fig. 4a–d). Additionally, cells assigned to CD subclone exhibited separation from the parental ABCD clone (Supplementary Fig. 3c). We repeated this analysis using t-SNE^31^, another dimensionality reduction technique. A subset of the cells assigned to the ancestral clone, and cells assigned to the EF clone, were well separated (Supplementary Fig. 3d). The observation of cluster-specific phenotypes, obtained through two independent methods, provides biological evidence of the capacity of PhylEx for phylo-phenotypic analysis.

We next sought to explore the relationship between pseudo-time trajectories and evolutionary history. Pseudo-time is a popular approach for looking at dynamic changes in gene expression over time. It was first applied in developmental biology studies^32^, but is increasingly being used in cancer studies^33^. An open question in the cancer context is whether pseudo-time trajectories reflect evolutionary history. As pseudo-time analysis is based purely on gene expression, this is not guaranteed. We applied the pseudo-time method Slingshot^34^ on the 2-dimensional representation obtained by ZINB-WaVE with the cells clustered using (i) PhylEx and (ii) mclust based on gene expression data^35^. Trajectories inferred by slingshot did not reflect the evolutionary histories: (i) the parent-child clones ABCD and CD appear as siblings (Fig. 3c) and (ii) the gene expression based clustering using mclust deviated significantly from the DLP cancer clones (Fig. 3d). These results suggest that phylo-phenotypic analysis will lead to accurate interpretations of the scRNA-seq data than ones based purely on gene expression and that trajectory analysis may not reflect evolutionary history of cancer.

We performed differential gene expression analysis (DGE) using edgeR^36,37^ to compare the three major clones: the Ancestral clone, the ABCD clone (primary tumor), and the EF clone (relapse tumor). The ancestral clone is represented by the cells assigned to the root node (161 cells), the ABCD clone is represented by the cells assigned to the left child of the root and its descendants (152 cells), and the EF clone is represented by the cells assigned to the right child of the root (47 cells) in the tree given in Fig. 3b. The resulting volcano plots reveal an abundance of differentially expressed genes between the Ancestral/ABCD dominant in the primary tumor, and the EF clone dominant in the relapse (Fig. 3e, f). There appears to be an evidence of immuno-editing in the relapse clone, manifested by a substantial number of down-regulated immune system genes. To verify this, we performed gene set enrichment analysis (GSEA) using Correlation Adjusted MEan RAnk gene set test available in limma package^38,39^ on the set MSigDB C5 (gene ontology)^40^. Several pathways related to the immune system were significantly down-regulated in the EF clone compared to the ABCD clone (Table 2 and Supplementary Table 1) suggesting evasion of immune surveillance as the primary relapse mechanism.

**Table 2:** Gene set enrichment analysis results comparing the ABCD clone to the EF clone

Gene ontology	*P*-value	FDR
MHC protein complex	4.85e-15	1.32e-11
Antigen processing and presentation of endogenous antigen	6.40e-15	1.32e-11
MHC class I protein complex	6.57e-14	8.08e-11
Response to type I interferon	2.86e-11	1.60e-08
Antigen processing and presentation of endogenous peptide antigen	2.61e-10	1.14e-07
Positive regulation of T cell mediated cytotoxicity	7.96e-09	2.51e-06
Interferon Gamma mediated signaling pathway	2.08e-08	5.72e-06
Regulation of T cell mediated cytotoxicity	2.37e-08	6.07e-06
Positive regulation of antigen processing and presentation	4.99e-07	9.59e-05
Detection of other organism	6.87e-07	1.28e-04

Top 10 most significantly down regulated pathways are shown (first column) along with *p*-value (middle column) and false discovery rates (third column). The Correlation Adjusted MEan RAnk gene set test is used, which performs a 2-sided test and uses Benjamini–Hochberg algorithm to produce false discovery rate (FDR) accounting for multiple comparisons^38^.

Taken together, these results demonstrate that insights obtained by comparing expression profiles in the context of PhylEx clones provide the capacity for phylo-phenotypic analysis which can be used to dissect the tumor gene expression patterns beyond what is possible with current single-cell expression analysis methods.

### Comparison of 10X and Smart-Seq3 scRNA-seq for clonal tree reconstruction

Next, we investigate the applicability of PhylEx on widely available 10X Genomics scRNA-seq data (referred to as 10X for brevity). Smart-Seq3, like its predecessor Smart-Seq2, is a plate-based, full-length transcript sequencing technology offering improved sensitivity to detect transcripts over its predecessor^29^. 10X on the other hand is a droplet based technology, which allows sequencing of large number of cells. While a comprehensive study comparing Smart-Seq3 to 10X is unavailable, the general understanding is that Smart-Seq3 offers better coverage and possibly depth on a smaller number of cells and 10X allows sequencing of a much larger number of cells at lower coverage^41,42^.

We obtained a total of 6616 cells sequenced using 10X 3′ sequencing of the HGSOC cell-lines. We computed sample statistics on the coverage of mutations across cells where we define a cell to cover a mutation at a loci if the read count at the loci contains at least one variant read. On average, cells sequenced using 10X platform had coverage of mutations for 0.4527 loci with the median of 0; in comparison, a cell acquired using Smart-Seq3 had coverage of mutations for 3.253 loci with median of 3 (Table 3). The low mutation coverage is a direct consequence of shallow read depth (Supplementary Fig. 8a, c) and the 3′ bias of the 10X data. The average depth at any given loci for 10X data was 1.403, conditional on having a minimum of one read. The average variant depth, defined as the number of reads mapping to the variant allele at a loci was 0.1427 conditional on the loci being expressed. With shallow depth, combined with possibility of mono-allelic expression, detecting mutations using 10X 3’ sequencing or similar approaches is challenging. In contrast, the Smart-Seq3 mean total depth was 19.37 and mean variant depth was 2.962.

**Table 3:** Coverage statistics for 10X vs Smart-Seq3 on HGSOC data

Method	1st Quantile	Median	Mean	3rd Quantile	Max
Smart-Seq3	1	3	3.253	5	14
10X	0	0	0.4527	1	4

Source data are provided as a Source Data file.

We compared PhylEx supplied with 10X scRNA-seq data to the bulk deconvolution methods TSSB and Canopy. We identified 93 SNVs for analysis and 540 cells that harbored variant reads on at least one of these SNVs using a filtering strategy similar to that applied to the Smart-Seq3 data (see “Methods” section). PhylEx outperformed the bulk deconvolution methods (Tables 4); however, the improvement in performance was not as significant as when supplied with Smart-Seq3 scRNA-seq data (Table 1). As PhylEx relies on co-occurrence of mutations to resolve temporal ordering of mutations as well as branching, it is critical that cells have as high coverage to achieve good performance. Computing the statistic on the selected 540 10X cells, we found that 428 cells had coverage of 2 mutations, 99 cells had coverage of 3 mutations, and 13 cells with coverage of 4 mutations. However, many of these had shallow coverage – once we restricted the definition of coverage to include at least two variant reads, the coverage statistic were 399 cells with 0 coverage, 129 with 1 mutation, and 11 cells with 2 mutations, and only 1 cell with 3 mutations.

**Table 4:** Performance metric comparing PhylEx on HGSOC data supplied with 10X scRNA-seq data to bulk-based deconvolution methods

Method	V-Measure	Adj. Rand Index	Adj. Mut Info	Anc. Recon Err
Canopy	0.305	0.176	0.168	0.245
TSSB	0.203 ± 0.0431	0.154 ± 0.0490	0.156 ± 0.0495	0.238 ± 0.0252
PhylEx	**0.360** ± **0.0418**	**0.206** ± **0.0323**	**0.266** ± **0.0376**	**0.233** ± **0.0129**

Used 20 runs for PhylEx and TSSB. Canopy was executed with four MCMC chains. The first column lists the name of the methods. The second to fourth columns are clustering metrics used for comparison. The last column is the ancestral reconstruction error metric. The boldface indicates the best performing method. Source data are provided as a Source Data file.

We have conducted simulation study to further corroborate our findings. We measured the performance of PhylEx on simulated scRNA-seq dataset with the coverage probability at {0.1, 0.05, 0.02} on a binary tree and bulk data generated with copy number variation using birth-death process (Supplementary Section 2.2-2.3). As expected, the performance improved as the coverage increased (Supplementary Fig. 8d, e). Note that at 0.02, we have very few cells which co-express variants (Supplementary Fig. 8f) and hence, the performance of PhylEx is indistinguishable from bulk deconvolution methods. These results suggest that using full-length transcript sequencing and higher sequencing depth can dramatically improve the clonal reconstruction accuracy. Note that our study involving 10X 3′ technology elected shallow sequencing depth to accommodate sequencing of thousands of cells. We expect coverage of mutation and PhylEx’s performance to improve at greater sequencing depth.

### Deciphering phenotypic evolution in HER2+ breast cancer

**Fig. 4:**
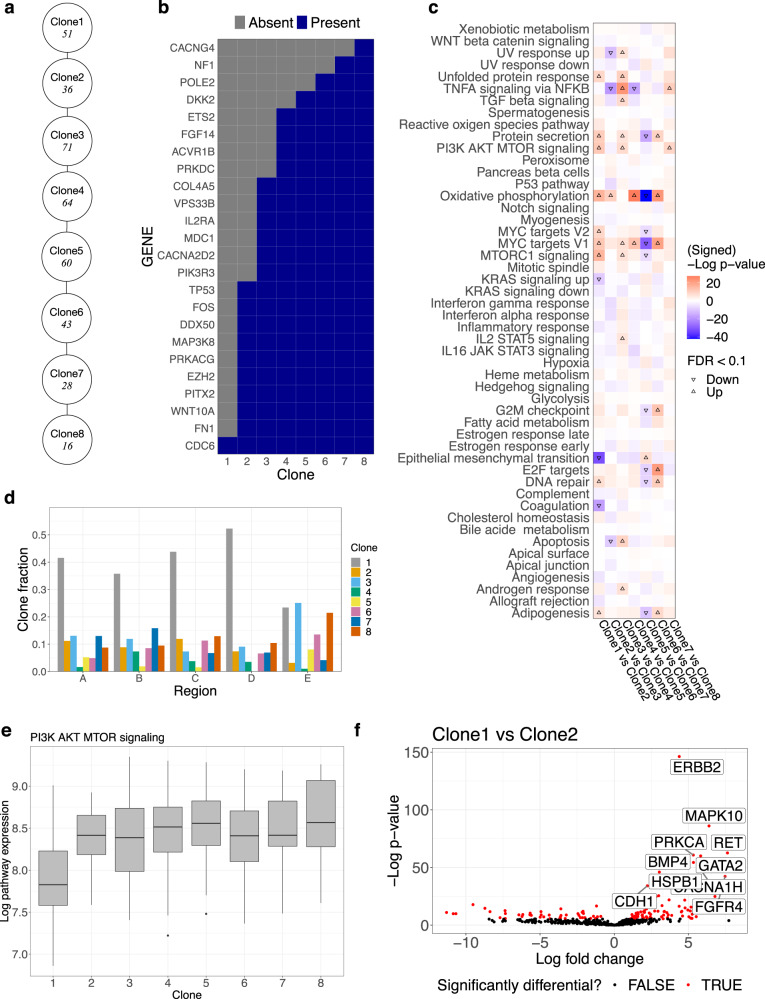
Multi-region HER2+ breast cancer analysis. **a** PhylEx inferred tree with the number of cells assigned to each clone shown under the clone label. **b** Mutation absence/presence heatmap. **c** Heatmap of gene set enrichment analysis on Hallmark pathways to compare parent-child clones. **d** Clone (cellular) fraction plot for each clone by region. **e** Box-plot of expression levels for PI3K AKT MTOR signaling pathway by clone; the 1st, 2nd, and 3rd quantiles are shown with the top (bottom) whisker indicates the maximal point no further than 1.5 × IQR from the third (first) quantile. **f** Differential gene expression analysis to compare progenitor cells assigned to Clone 2 to the cells to Clone 1. Source data are provided as a Source Data file.

We generated Smart-Seq3 scRNA-seq and bulk whole-exome DNA sequencing data for five spatially distinct regions of an untreated HER2+ breast cancer tumor. We applied PhylEx to 369 cells and 418 SNVs that were available after pre-processing. The PhylEx MAP tree was a linear expansion, i.e., a path (Fig. 4a), after restriction to clones that contained more than 1 SNV and at least one cell assigned (Supplementary Fig. 6b). We also applied TSSB on the data without scRNA-seq data. The TSSB tree infers a linear expansion until the end where we see two clones branching (Clones 5 and 6 in Supplementary Fig. 6a). After assigning cells to this tree, we see that mutual exclusivity of the mutations are violated (Supplementary Fig. 6d). In contrast, we see that cells assigned on PhylEx tree form clear partitions with minimal violation (Supplementary Fig. 6e); this figure shows single-cell data support for the linear evolution.

The clone fraction appeared to be well-mixed in each region (Fig. 4d). The clone fraction of regions D, E differed from the other regions; this is perhaps explained by the fact that these regions were relatively far away from the other regions (Supplementary Fig. 5g).

We retrieved the NanoString PanCancer human pathway panel gene list of 770 curated genes (NanoString Technologies, Seattle, WA) for the downstream analysis. Focusing on this set of genes helps to identify driver mutations for each clone. Among the SNVs used in our analysis, 24 overlapped the NanoString list (Fig. 4b and Supplementary Table 2). We identified a mutation in *CDC6* in the progenitor clone (Clone 1), implicating changes to the cell replication mechanism, and identified a mutation in *TP53* and *MAP3K8* in Clone 2, hinting at the proliferation of cancer beginning at Clone 2. In Clone 3, we noted mutations to genes involved in PI3K and MAPK pathways (*PIK3R3*, *CACNA2D2*) and to *MDC1* (DNA repair). Clone 4 appears to be characterized by changes to the RAS pathway as evidenced by mutations to *ETS2*. Overall, the clonal tree provides a vital context in which to analyze and inspect mutations in cancer.

We performed gene set enrichment analysis on the MSigDB Hallmark gene sets to compare the parent-child clones (Fig. 4c). GSEA revealed a significant increase of PI3K AKT MTOR signaling pathway expression in Clone 2 compared to Clone 1. The PI3K AKT MTOR pathway is a commonly activated therapeutic target in breast cancer^43^. An in-depth inspection of the expression revealed an upregulation of PI3K AKT MTOR signaling pathway in all clones descending from Clone 1 (Fig. 4e). We then performed DGE to compare the clones (Fig. 4f and Supplementary Fig. 5a–f). We confirmed an overexpression of *ERBB2* in Clone 2 compared to Clone 1 (FDR < 0.1). Clone 1 had a mutation in CDC6 and only two other mutations, perhaps indicating that its cells more closely resemble normal cells than the cancer cells.

Overall, the PhylEx analysis identifies the driver mutations (Fig. 4b), elucidates spatial distribution of the clones (Fig. 4d), and facilitates a downstream analysis of scRNA expression data that sheds light on the clones’ functional characteristics (Fig. 4c, e, f).

## Discussion

In this work, we have presented PhylEx, for integrating bulk genomic and single-cell transcriptomic data to reconstruct clonal trees, which paves the road for characterizing the functional state of individual clones via phylo-phenotypic analysis. We have shown how PhylEx enhances downstream analysis by providing a clonal tree and the opportunity to compare the clones’ functional states – revealing the interplay between the evolutionary process and the clones’ phenotypes.

We established that specialized methods for integrating bulk with single-cell transcriptomics are necessary. By modeling read counts, PhylEx bypasses the need for performing cell genotyping and hence, avoids compounding of errors stemming from dichotomizing counts into binary values. PhylEx, using only a single region bulk sequencing combined with scRNA-seq, outperforms state-of-the-art bulk-based methods supplied with multi-region data. We expect these findings to shift the paradigm from multi-region sequencing to single-region sequencing accompanied by scRNA-seq. This approach will simultaneously reduce the effort required for data acquisition, improve the accuracy of the clonal reconstruction, and allow for functional analysis of individual clones. Moreover, many researchers will realize that the single-cell RNA data they already possess should be exploited for clonal analysis or, even, to perform supplementary single-cell RNA sequencing for this purpose. With the prevalence of bulk DNA sequencing and rapidly growing studies conducting scRNA-seq, we expect that PhylEx will prove profitable to cancer researchers studying the functional implications of cancer evolution.

Furthermore, PhylEx opens the avenue for future extension to characterize clones by somatic mutations as well as copy number profiles. In particular, inferring subclonal copy numbers is inherently challenging to achieve using only the bulk sequencing data. It is currently feasible using specialized single-cell sequencing techniques such as DLP^19,28,44^. There exist methods that perform copy number inference from scRNA-seq data such as InferCNV, HoneyBADGER, and CopyKAT^45–47^; however, these methods do not consider copy number variation in the context of evolution. For example, CopyKAT, relies on hierarchical clustering on the expression data. While InferCNV and HoneyBADGER allow subclonal copy number inference, they are limited to bifurcating trees. With evidence for multifurcation in cancer evolution (e.g., ref. ^25^) as well as linear evolution^48^, coupled with a lack of resolution to detecting binary branching from scRNA-seq data, this is potentially a severe limitation. The performances of these methods also depend on having a set of normal reference cells as CNV inference from scRNA-seq data require reference expression levels of the normal cells. As such, CNV clones did not have high concordance with SNV clones when applied to HER2+ scRNA-seq data (Supplementary Fig. 6c, f) where we did not have normal reference cells.

PhylEx, as is the case with other methods, has limitations. A full-length single-cell transcript sequencing technology with sufficient coverage and depth of sequencing is necessary to attain accurate inference of clonal trees. Although the algorithmic complexity is linear in the number of cells, it also depends on the size of the clonal tree. As the size of the clonal tree may grow for cancers with complex evolutionary process, we recommend the users to carefully select SNVs to include in the analysis, e.g., tumor suppressor genes, oncogenes, and deleterious mutations. We noted that Cardelino’s mapping algorithm performs slightly better than PhylEx as shown in Supplementary Fig. 2; therefore, recommended workflow is to infer clonal tree using PhylEx and map cells on PhylEx clones using Cardelino. Finally, PhylEx uses copy number profiles inferred from bulk genomics data. While estimating CNV from bulk is a well-established technology and our approach can mitigate the effects of approximation error via marginalization (Methods), PhylEx can benefit from integrating copy number information in the bulk as well as in scRNA-seq data. We identify that the next challenge is to perform joint inference of clonal tree, clonal genotypes including SNVs and copy numbers via integration of scRNA-seq with bulk DNA-seq data. This calls for a statistical model that captures the dependence between the copy numbers and the observed read counts in the scRNA-seq as well as the bulk data, and tractable computational algorithms to cope with potentially large computational cost associated with hidden Markov model operating over tree on the evolving copy number profiles. PhylEx represents an important first step and a substantial progress in reconstructing the entire evolutionary trajectory of cancer towards accomplishing this goal.

## Methods

### Ethics statement on collection of clinical material for breast cancer samples

Fresh primary tumor resections were obtained from a breast cancer patient at Karolinska University Hospital and Stockholm South General Hospital. Experimental procedures and protocols were approved by the regional ethics review board (Etikprövningsnämnden) in Stockholm, with reference numbers 2016/957-31 and 2017/742-32. Biobank approval was obtained from the Stockholm medical biobank. Before surgery, informed consent in accordance with the Declaration of Helsinki was given to the patient for signature. The patient was not compensated since our study did not include any extra steps other than the standard treatment procedures for the disease.

### Whole-exome sequencing for breast cancer samples

Tumor resections and matching dermal biopsies from 4 individual breast cancer patients were freshly collected. Tissues were manually homogenized and genomic DNA samples were isolated by using the QIAamp DNA mini kit (QIAGEN). The library was prepared by using Twist Bioscience Human Core Exome kit (Twist Bioscience) according to the manufacture protocol. The bulk DNA samples were then sequenced in a S4 flow cell lane by the NovaSeq 6000 platform (Illumina) at the National Genomics Infrastructure, Science for Life Laboratory, Uppsala.

### Breast cancer sample preparation for single-cell RNA sequencing

Tissues were homogenized and cells were released by using the gentleMACS™ Octo Dissociator with Heaters and the human tumor dissociation kit (both from Miltenyi Biotec), according to the manufacturer protocols. Afterwards, the cells were washed two times with F12-DMEM medium (Gibco) and collected by centrifugation at 300g for 5 minutes. The single-cell suspensions were further generated by passing the resuspended cells through the 70 mm cell strainers. The single cell suspensions were then further stained with the Zombie Aqua Fixable viability dye (1:100, Biolegend, 423101) at room temperature for 20 min, then washed with phosphate-buffered saline (PBS). The cells were incubated with Human TruStain Fc block (1:100, Biolegend, 422302) for 10 min to limit unspecific antibody binding, then stained for 20 min with anti-EPCAM (1:40, Biolegend, 324206) and anti-CD45 (1:40, Biolegend, 304021) in FACS buffer (PBS + 0.5% Bovine Serum Albumin). The cells were subsequently washed and resuspended in FACS buffer. Fluorescence-activated cell sorting (FACS) using an influx flow cytometer (BD Biosciences) was performed to sort live EPCAM+CD45- single-cells into 384 well plates for Smart-Seq3 analysis. The list of antibodies is provided in Supplementary Data 2.

### Ovarian cancer cell lines preparation for Smart-Seq3

Culture of ovarian cancer cell lines OV2295, TOV2295, and OV2295R cells were cultured in a 1:1 mix of Media 199 (Sigma Aldrich) and MCDB 105 (Sigma Aldrich) supplemented with 10% FBS in a humidified environment at 37C. For single-cell RNA sequencing, all cells used in this study were sorted on a BD Influx into 384 well plates using index-sorting and single-cell purity mode directly into lysis buffer(6,67% Polyethylene Glycol, 0.1% Triton X-100, RNAse Inhibitor (Takara), dNTPs (0.67 mM/each), and Oligo-dT (0.67uM)). Sorted plates were stored at −80 °C and thawed immediately prior to library generation. The cell line originates from ref. ^27^.

### Smart-Seq3 library preparation and sequencing

For single-cell RNAseq libraries, the Smart-Seq3 method was used according to the published protocol (PMID: 32518404). In brief, plates were quickly centrifuged before reverse transcription (25 mM Tris-HCl pH 8.3 (Sigma), 30 mM NaCl (ThermoFisher), 2.5 mM MgCl2 (ThermoFisher), 1 mM GTP (ThermoFisher), 8 mM DTT (ThermoFisher), 0.5 μ/μl RNase inhibitor (Takara), 2 μM TSO (IDT), 2 μ/μl Maxima H-minus reverse transcriptase (ThermoFisher)), and amplified using KAPA HiFI Hotstart polymerase (Roche) to generate full-length cDNA libraries (22 cycles PCR). Final library concentrations were determined and normalized for each cell using Picogreen. Diluted cDNA of 100 pg per sample was used for tagmentation (Nextera Library Preparation Kit, Illumina, ATM at 0.1 μL per cell). The final samples were analyzed using a Bioanalyzer (Hi-Sensitivity Kit, Agilent) and sent for sequencing on a Novaseq S Prime lane, PE 2x150bp (Illumina). Library quality was compared to index sorting results to confirm that negative wells yielded low complexity libraries. The list of oligonucleotides are available at 10.17504/protocols.io.bcq4ivyw and provided as Supplementary Data 3.

### PhylEx probabilistic model

PhylEx performs Bayesian posterior inference over the clonal tree, assignment of SNVs to clones, cellular prevalences by integrating bulk DNA- and scRNA-seq data. The graphical model for PhylEx is provided in Supplementary Fig. 9 and the table of notation along with brief description for each variable is given in Supplementary Table 4.

#### Model overview

We define the latent clonal tree *T* as a rooted tree with the nodeset denoted by *V*. The nodeset represents the set of clones; we will use the term node and clone interchangeably. The root node *r* represents healthy cells and has exactly one child; the lone child of the root represents the cancer progenitor clone. Each non-root node *v* has one parent, denoted *ρ*(*v*) but each non-root node may have any number of children (zero or more), the set of children of *v* is denoted *κ*(*v*). We achieve flexibility in modeling the number of children by using tree-structured stick-breaking process (TSSB) prior^20^, which is a prior over arbitrary tree depth and width, particularly useful in modeling clonal trees where the number of branching events is unknown in advance. We denote the number of SNVs under consideration by *N*. The clonal membership of SNVs is represented by **z** = (*z*_1_, …, *z*_*N*_), where *z*_*n*_ ∈ *V* for *n* = 1, …, *N*. The aforementioned TSSB defines a joint distribution over *T*, **z**, which we denote *P*_0_(**z**, *T*).

Each node of the tree is associated with cellular prevalence parameter denoted $\phi={({\phi }_{v})}_{v\in V}$. Note that we can associate cellular prevalence parameter to each SNV *n* = 1, ..., *N* as follows: ${\phi }_{n}={\phi }_{{z}_{n}}$ (i.e., the cellular prevalence parameters are shared by SNVs given the latent clonal membership). The prior distribution over the cellular prevalences is given by hierarchical priors conditional on *T*, **z**, first introduced in PhyloSub and PhyloWGS^6,7^, which we denote as *P*_0_(*ϕ*∣**z**, *T*). The hierarhical priors enforce the following restrictions on the cellular prevalence parameters: (1) ∑_*u*∈*κ*(*v*)_ *ϕ*_*u*_ ≤ *ϕ*_*v*_ and (2) 0 ≤ *ϕ*_*v*_ ≤ 1. The cellular prevalence of a clone represents the proportion of cell population that inherit the genomic profiles of the clone (in our case, SNVs). Therefore, one important property that must be satisfied is the sum of the cellular prevalence of the descendants of a clone *v* to not exceed its own cellular prevalence *ϕ*_*v*_. The first restriction enforces this property. The second restriction enforces the fact that we are dealing with the proportion of cell population (i.e., a number between 0 and 1).

From the bulk data, we assume clonal copy number information is available along with the number of reads mapping to the variant and reference alleles. The clonal copy number can be obtained using a wide range of public software (e.g., refs. ^49–52^); we use TitanCNA^51^, from which we obtain major and minor copy numbers, (*M*_*n*_, *m*_*n*_), for each SNV, *n* = 1, ..., *N*. Hence, we denote the bulk data by ${{{{{{{\boldsymbol{B}}}}}}}}={\{({b}_{n},\,{d}_{n},\,{M}_{n},\,{m}_{n})\}}_{n=1}^{N}$, where *b*_*n*_, *d*_*n*_ denote the variant reads and read depth at locus *n*. The scRNA-seq data is denoted by ${{{{{{{\boldsymbol{S}}}}}}}}={\{{\{{b}_{c,n},\,{d}_{c,n}\}}_{n=1}^{N}\}_{c=1}^{C}}$, where *C* denotes the number of cells and *b*_*c*,*n*_, *d*_*c*,*n*_ denote the variant reads and read depth at locus *n* for cell *c*.

The likelihood of the bulk and single-cell data is assumed to be conditionally independent given *T*, **z**, *ϕ*:

1 $$\ell ({{{{{{{\boldsymbol{B}}}}}}}},\,{{{{{{{\boldsymbol{S}}}}}}}}|T,\,{{{{{{{\bf{z}}}}}}}},\,\phi )=\ell ({{{{{{{\boldsymbol{B}}}}}}}}|T,\,{{{{{{{\bf{z}}}}}}}},\,\phi )\ell ({{{{{{{\boldsymbol{S}}}}}}}}|T,\,{{{{{{{\bf{z}}}}}}}},\,\phi ).$$

The posterior distribution over the latent variables, *T*, **z**, *ϕ* is expressed in terms of this likelihood and the prior distributions as follows:

2 $$P(T,\,{{{{{{{\bf{z}}}}}}}},\,\phi|{{{{{{{\boldsymbol{B}}}}}}}},\,{{{{{{{\boldsymbol{S}}}}}}}})\propto \ell ({{{{{{{\boldsymbol{B}}}}}}}}|T,\,{{{{{{{\bf{z}}}}}}}},\,\phi )\ell ({{{{{{{\boldsymbol{S}}}}}}}}|T,\,{{{{{{{\bf{z}}}}}}}},\,\phi ){P}_{0}(\phi|T,\,{{{{{{{\bf{z}}}}}}}}){P}_{0}({{{{{{{\bf{z}}}}}}}},\,T),$$

where *P*_0_(**z**, *T*) is given by tree-structured stick-breaking process (TSSB) prior^20^ and *P*_0_(*ϕ*∣*T*, **z**) is adopted from ref. ^6^.

#### Prior distributions

The tree structured stick breaking (TSSB) process is a Bayesian non-parametric prior defined on infinite trees where a unit length stick is recursively partitioned by nodes of the tree. The TSSB process has proven to be useful in cancer phylogenetics, e.g., refs. ^6–8^. In PhylEx, TSSB is used as a prior distribution over the SNV assignment and the tree topology, *P*_0_(*T*, **z**∣*λ*_0_, *λ*, *γ*) with hyperparameters *λ*_0_ > 0, *λ* ∈ (0, 1], *γ* > 0. We briefly summarize the role of hyperparameters on the shape of the tree topology as described in ref. ^20^.

TSSB prior defines partition of unit length stick to the nodes of the tree, where this partitioning is determined by *ν*-sticks and *ψ*-sticks. The *ν* sticks are used to allocate the size of the stick assigned to the nodes while the *ψ* sticks determine the size of the sticks to be allocated to the children. Let *υ*_*u*_ denote the portion of the stick available to be broken up by node *u* and *π*_*u*_ denote the portion of the unit length stick assigned to node *u*. To determine *π*_*u*_, we first sample *ν*_*u*_ ~ Beta(1, *λ*_0_*λ*^∣*u*∣^), where ∣*u*∣ denotes the height of node *u* in the tree. Then, we set *π*_*u*_ = *ν*_*u*_*υ*_*u*_ to determine the portion of the unit-stick assigned to node *u*. The remaining stick, (1 − *π*_*u*_)*υ*_*u*_, is allocated to the children of *u* in the following manner: sample *ψ*_*u*,*k*_ ~ Beta(1, *γ*) for children *k* = 1, 2, ... of *u* and then setting *υ*_*u*,*k*_ = (1−*π*_*u*_)*υ*_*u*_*ψ*_*k*_ ∏_*j*<*k*_(1−*ψ*_*u*,*j*_).

The prior probability of an SNV being assigned to node *u* is proportional to *π*_*u*_, hence, the height of the tree is closely related to the size of the *ν*-stick. The larger the *ν*-sticks broken by an ancestral nodes, smaller the stick length available for the descendant nodes. Therefore, the hyperparameters *λ*_0_, *λ* govern the height of the tree. It is straightforward to see that the width of the tree depends on *γ*. We found that setting *γ* ≤ 1 makes the most sense for cancer phylogenetics applications since the number of branches in a clonal tree is relatively small; note that *γ* = 1 ⇒ *ψ*_*u*,*k*_ ~ Uniform(0, 1). We provide details on setting the hyperparameters to control the TSSB parameters in Supplementary Section 3.

The prior distribution on the cellular prevalences, *P*_0_(*ϕ*∣**z**, *T*), is adopted from ref. ^6^. In essence, this amounts to converting the cellular prevalences to clone fractions,

3 $${\eta }_{u}={\phi }_{u}-\mathop{\sum}\limits_{{u}^{{\prime} }\in \kappa (u)}{\phi }_{{u}^{{\prime} }}.$$

Note that ∑_*u*_*η*_*u*_ = 1 for a fixed tree *T* and hence, we can place a Dirichlet distribution on *η*_*u*_ as a prior distribution, conditioned on tree *T*.

#### Modeling the bulk DNA-seq data

The bulk data likelihood assumes site independence conditional on *T*, **z**:

4 $$\ell ({{{{{{{\boldsymbol{B}}}}}}}}|T,\,{{{{{{{\bf{z}}}}}}}},\,\phi )\propto \mathop{\prod }\limits_{n=1}^{N}P({b}_{n}|T,\,{{{{{{{\bf{z}}}}}}}},\,\phi,\,{d}_{n},\,{M}_{n},\,{m}_{n}).$$

All possible copy number profiles is marginalized to compute the likelihood of the observed reads

5 $$P({b}_{n}|T,\,{{{{{{{\bf{z}}}}}}}},\,\phi,\,{d}_{n},\,{M}_{n},\,{m}_{n})=\mathop{\sum}\limits_{{g}_{n}\in {{{{{{{\mathcal{G}}}}}}}}({M}_{n},\,{m}_{n})}P({b}_{n}|{d}_{n},\,{g}_{n},\,{\phi }_{{z}_{n}})P({g}_{n}|{M}_{n},\,{m}_{n}),$$

where ${{{{{{{\mathcal{G}}}}}}}}({M}_{n},\,{m}_{n})$ are possible genotypes compatible with a given major and minor copy number profile. We use uniform prior over all possible genotypes, i.e., $P({g}_{n})=1/|{{{{{{{\mathcal{G}}}}}}}}({M}_{n},\,{m}_{n})|$. A detailed description of the marginalization process over the genotypes is provided in the Supplementary Text in ref. ^5^, under section heading *The PyClone model description*; we also provide an example to illustrate the marginalization process in the Supplementary Section 1.1. The probability distribution for the observed variant read at each site is given by Binomial distribution:

6 $${b}_{n}|{d}_{n},\,{g}_{n},\,{\phi }_{{z}_{n}} \sim \,{{\mbox{Binomial}}}\,({d}_{n},\,\theta ({g}_{n},\,{\phi }_{{z}_{n}},\,\epsilon )),$$

with $\theta ({g}_{n},\,{\phi }_{{z}_{n}},\,\epsilon )$ being the probability of success given as a function of the genotype, cellular prevalence of clone *z*_*n*_, and sequencing error probability, *ϵ*. Note that ${\phi }_{{z}_{n}}$ is the cellular prevalence of the clone where the *n*-th SNV is assigned since *z*_*n*_ ∈ *V* denotes the assignment of SNV *n* to a clone (recall that all SNVs assigned to the same clone share the same cellular prevalence). Letting *v*(*g*), *c*(*g*) be the number of variant copies and total copy numbers for a genotype *g*, the success probability is given by,

7 $$\theta ({g}_{n},\,{\phi }_{{z}_{n}},\,\epsilon )=\left\{\begin{array}{ll}\epsilon \hfill&\,{{\mbox{if}}}\,\,v({g}_{n})=0\hfill\\ {\phi }_{{z}_{n}}(1-\epsilon )+(1-{\phi }_{{z}_{n}})\epsilon &\,{{\mbox{if}}}\,\,v({g}_{n})=c({g}_{n})\\ {\phi }_{{z}_{n}}\frac{v({g}_{n})}{c({g}_{n})}+(1-{\phi }_{{z}_{n}})\epsilon \hfill&\,{{\mbox{otherwise}}}\,.\hfill\end{array}\right.$$

#### Modeling the scRNA-seq data

We assume that the scRNA-seq likelihood is conditionally independent over cell and locus given *T*, **z** and cell-to-clone membership, ${{{{{{{\boldsymbol{\zeta }}}}}}}}={({\zeta }_{c})}_{c=1}^{C}$:

8 $$\ell ({{{{{{{\boldsymbol{S}}}}}}}}|T,\,{{{{{{{\bf{z}}}}}}}},\,\phi,\,{{{{{{{\boldsymbol{\zeta }}}}}}}})\propto \mathop{\prod }\limits_{c=1}^{C}\mathop{\prod }\limits_{n=1}^{N}P({b}_{c,n}|T,\,{{{{{{{\bf{z}}}}}}}},\,{\zeta }_{c},\,{d}_{c,n}).$$

The cell-to-clone membership variable completely determines the SNVs harbored by cells: for a cell assigned to node *u*, it inherits all of the SNVs assigned to ancestral nodes of *u*. We denote the mutation status of cell *c* for locus *n* by *μ*_*c*,*n*_ ∈ {0, 1}, which can be seen as a function of *T*, **z**, *ζ*_*c*_ (i.e., can be read off from these quantities).

As a first step to modeling the number of variant reads, we plotted the histogram of the ratio of variant reads to depth over all sites and cells i.e., *b*_*c*,*n*_/*d*_*c*,*n*_ for HGSOC data (Supplementary Fig. 7d) and HER2+ scRNA-seq data (Supplementary Fig. 7e). These plots clearly depict the mono-allelic nature of the expression data, with inflation at 0 and 1. Note that zero-inflation is pronounced because there are two cases that can lead to non-expression of variant: (1) mono-allelic expression of reference allele and (2) absence of variant allele or no mutation (Supplementary Fig. 7h). We plotted the bi-allelic sites by selecting a subset of the data such that *b*_*c*,*n*_ > 0 and *b*_*c*,*n*_/*d*_*c*,*n*_ < 1 (Supplementary Fig. 7f, g). These plots point towards a mixture of distributions as a suitable model for the scRNA-seq read counts as the mixture can account for stochastic nature of scRNA-seq, in particular, we need one distribution to model mono-allelic expression and another for bi-allelic distribution. Similar techniques are employed in refs. ^18,24,53^.

For cell *c* that does not harbor mutation at locus *n*, we have a simple error model:

9 $${b}_{c,n}|{d}_{c,n},\,\epsilon \sim \,{{\mbox{BetaBinomial}}}\,({d}_{c,n},\,\epsilon,\,1-\epsilon ).$$

The error distribution uses sequencing error probability *ϵ* (Supplementary Fig. 7c). For cell *c* that harbors the mutation at locus *n*, we assume the following generative process:

10 $$	{\delta }_{c,n} \sim {{{{{{{\rm{Bernoulli}}}}}}}}({\delta }_{n}^{0})\\ 	{\chi }_{c,n}|{\delta }_{c,n} \sim {\delta }_{c,n}{{{{{{{\rm{Beta}}}}}}}}({\alpha }_{n},\,{\beta }_{n})+(1-{\delta }_{c,n}){{{{{{{\rm{Beta}}}}}}}}({\alpha }_{0},\,{\beta }_{0})\\ 	{b}_{c,n}|{d}_{c,n},\,{\chi }_{c,n} \sim {{{{{{{\rm{Binomial}}}}}}}}({d}_{c,n},\,{\chi }_{c,n})$$

where ${\delta }_{n}^{0}$ is the prior probability of bi-allelic expression at locus *n* and *δ*_*c*,*n*_ ∈ {0, 1} is an indicator variable denoting bi-allelic (*δ*_*c*,*n*_ = 1) or mono-allelic (*δ*_*c*,*n*_ = 0) expression, and *χ*_*c*,*n*_ as the probability of expressing the variant allele for cell *c* at locus *n*. The parameters of the Beta distribution, *α*_0_, *β*_0_, *α*_*n*_, *β*_*n*_ are hyperparameters of the model. For small values of *α*_0_, *β*_0_, the Beta distribution places most of the probability mass at the two ends as shown in Supplementary Fig. 7a, b, making it suitable for modeling mono-allelic distribution; setting *α*_0_ = *β*_0_ makes the distribution symmetric. We use *α*_0_ = *β*_0_ = 0.01 for HGSOC and HER2+ analysis and set ${\delta }_{n}^{0}=0.5$ for *n* = 1, ..., *N*. The parameters *α*_*n*_, *β*_*n*_ determine the levels of bi-allelic expression and are estimated as part of data pre-processing step (Supplementary Section 1.2). The above generative model can be combined into Beta-Binomial mixture so as to suppress explicit dependence on *χ*_*c*,*n*_:

11 $$	{b}_{c,n}|{d}_{c,n},\,{\delta }_{c,n},\,\epsilon \sim \\ 	\left\{\begin{array}{ll}(1-{\delta }_{c,n}){{{{{{{\rm{BetaBinomial}}}}}}}}({d}_{c,n},\,{\alpha }_{0},\,{\beta }_{0})+{\delta }_{c,n}{{{{{{{\rm{BetaBinomial}}}}}}}}({d}_{c,n},\,{\alpha }_{n},{\beta }_{n})&{{{{{{{\rm{if}}}}}}}}\,{\mu }_{c,n}=1\hfill\\ {{{{{{{\rm{BetaBinomial}}}}}}}}({d}_{c,n},\,\epsilon,\,1-\epsilon )\hfill&{{{{{{{\rm{otherwise}}}}}}}}.\end{array}\right.$$

In the computation of the likelihood, we marginalize out *δ*_*c*,*n*_ as well.

The prior probability of cell assignment to clone *u* can be given by the clone fraction, *η*_*u*_. However, such an assumption may not hold as cells with certain characteristics may be preferentially selected for sequencing. Therefore, we use Uniform distribution:

12 $$P({\zeta }_{c}|{{{{{{{\bf{z}}}}}}}},\,T,\,\phi )\propto 1.$$

In evaluating the single-cell component of the likelihood for a given tree, we marginalize over the cell-to-clone assignments,

13 $$\ell ({{{{{{{\boldsymbol{S}}}}}}}}|T,\,{{{{{{{\bf{z}}}}}}}},\,\phi )=\mathop{\prod }\limits_{c=1}^{C}\mathop{\sum}\limits_{{\zeta }_{c}}\mathop{\prod }\limits_{n=1}^{N}P({b}_{c,n}|T,\,{{{{{{{\bf{z}}}}}}}},\,{\zeta }_{c},\,{d}_{c,n})P({\zeta }_{c}).$$

### Runtime analysis

Inference is performed using slice sampling as described in ref. ^20^ and MH sampler is as described in ref. ^6^. One iteration considers re-assignment of each of the SNVs in some predetermined order much like Gibbs sampling. After re-assignment of all SNVs, MH sampler is invoked to update the cellular prevalences. As the original slice sampler only requires bulk likelihood computation, the runtime for re-assigning an SNV is *O*(1). One iteration of the slice sampler for PhylEx requires computation of bulk data likelihood as well as the single cell data likelihood. As we marginalize over the clone assignment of the single cells, the computational cost requires *O*(*C* ⋅ ∣*V*∣).

### Evaluation metrics

We used V-measure, adjusted rand index, and adjusted mutual information as implemented in scikit-learn (version 0.23.1)^54^. To evaluate the reconstruction accuracy, we use an ancestral reconstruction error, defined on a pair of SNVs as follows. For two nodes *u*, *v* in the tree, we say *u* < *v* to mean that *u* is ancestral to *v*. We can extend this definition to SNVs *i*, *j*. We will say *i* < *j* if and only if *i* is assigned to node *u* and *j* to *v* such that *u* < *v*. We formulate an ancestral matrix of dimension *N* × *N*, where the (*i*, *j*)-th is set to 1 if *i* < *j*. We denote the ancestral matrix for the ground truth SNV-to-clone assignment by A^*^, then we can compute the absolute error (AE) of an ancestral matrix *A* by summing over unique pairs:

14 $${{{{{{{\rm{AE}}}}}}}}({A}^{*},\,A)=\mathop{\sum}\limits_{(i,j)}|{A}_{i,j}^{*}-{A}_{i,j}|.$$

The mean absolute error is given by dividing AE by the number of unique pairs. We define the loss function on predicted mutation status of SNVs for cells as:

15 $$L({{{{{{{{\boldsymbol{\mu }}}}}}}}}_{c},\,{{{\hat{{{{{\boldsymbol{\mu }}}}}}}}}({\zeta }_{c},\,{{{{{{{\bf{z}}}}}}}},\,T))=\#[{{{{{{{{\boldsymbol{\mu }}}}}}}}}_{c}\,\ne \, {{{\hat{{{{{\boldsymbol{\mu }}}}}}}}}({\zeta }_{c},\,{{{{{{{\bf{z}}}}}}}},\,T)],$$

where ${{{{{{{{\boldsymbol{\mu }}}}}}}}}_{c}={({\mu }_{c,n})}_{n=1}^{N}$ is a vector of length *N* denoting the true mutation status for cell *c*, ${{{\hat{{{{{\boldsymbol{\mu }}}}}}}}}({\zeta }_{c},\,{{{{{{{\bf{z}}}}}}}},\,T)$ is a vector of length *N* denoting the predicted mutation status for cell *c*, and *#*[***a*** ≠ ***b***] denotes the number of entries where vectors ***a***, ***b*** disagree. The expected loss marginalizing over the cell-to-clone assignment is then defined as,

16 $${{\mathbb{E}}}_{{\zeta }_{c}}[L({{{{{{{{\boldsymbol{\mu }}}}}}}}}_{c},\, {{{\hat{{{{{\boldsymbol{\mu }}}}}}}}}({\zeta }_{c},\, {{{{{{{\bf{z}}}}}}}},\, T))]=\mathop{\sum}\limits_{v\in V}P({\zeta }_{c}=v)\times\#[{{{{{{{{\boldsymbol{\mu }}}}}}}}}_{c} \, \ne \, {{{\hat{{{{{\boldsymbol{\mu }}}}}}}}}({\zeta }_{c},{{{{{{{\bf{z}}}}}}}},\, T)].$$

### Processing of bulk DNA sequencing data

The bulk tumor and matching normal samples are to be pre-processed following standard guidelines as per GATK standard practice^55^. For variant calling, we used Strelka v2.9.2 and Mutect2 as part of GATK v4.1.4.0^56,57^. We processed the VCF file using vcfR v1.12.0^58^ to obtain for each SNV (i) position in the genome (loci), (ii) the variant and reference alleles, (iii) the number of reads mapping to variant and reference alleles. We used the PASS filter to select the high-confidence SNVs. PhylEx requires major and minor copy number profiles for the SNVs; we used TitanCNA v1.24.0^51^. Given these input data, we provide functionalities to prepare the input data for running PhylEx in the code repository: https://github.com/junseonghwan/PhylExAnalysis. We used Falcon v0.2^52^ to obtain copy number profiles needed for running Canopy^9^.

### Processing of single-cell RNA sequencing files

Individual fastq files for the cells are obtained using Illumina bcl2fq tool, then converted to ubam format with cell and UMI tags using a script that detect Smart-Seq 3 specific pattern at the beginning of reads with UMI. STAR v2.7.3^59^ with GRCh37 version of Human Genome and Ensembl version 75 annotations were used to align the reads^60^. UMI-tools v1.1.1^61^ was then used to correct the UMI and group UMI reads. An in-house script was used to intersect the reads with bam files and obtain read and UMI counts for each gene; we used Rsamtools v2.2.3^62^ to obtain the reads mapping to the variant and the reference alleles from the BAM file needed for running PhylEx and used Rsubread v2.0.1^63^ to generate feature counts for downstream gene expression analysis. For 10X Chromium data 10X Genomics CellRanger v6.0.1 was used to generate BAM files and the count matrix^64^.

### Data processing steps specific to the high-grade serous ovarian cancer cell-line

The pseudobulk DNA-seq data are obtained by combining scDNA data analyzed in^19^ using samtools v1.9^65^. The copy number profiles are obtained from the bulk samples using TitanCNA and the scRNA-seq is aligned using STAR aligner. A list of SNVs are provided in the data repository provided in ref. ^19^; therefore, we did not need to make variant calls. Among the list of SNVs provided in ref. ^19^, 634 SNVs were found to be exonic. We further filtered these set of SNVs using scRNA-seq data. For each loci *n* = 1, …, 634, we selected it for analysis if there were at least two cells such that *b*_*c*,*n*_ ≥ 2, which resulted in 67 SNVs. Including SNVs that do not have sufficient single-cell coverage does not help to evaluating different methods and their capacities for inferring the branching events and the ancestral relationship. We retrieved the reads at each of these 67 loci for each cell using Rsamtools v2.2.3. A similar approach was adopted for 10X analysis, however, due to shallow depth, we used *b*_*c*,*n*_ ≥ 1 as using a more stringent condition resulted in dropping most of the SNVs from the analysis.

### Software used for data analysis

We ran ddClone v0.2, B-SCITE v2.0, PhyloWGS v1.0, Canopy v1.3.0, and Cardelino v0.6.4 for benchmarking^7,9,15,16,18^. The downstream gene expression analyis was performed in R v3.6.3 (also tested on v4.0.3)^66^. We used biomaRt v2.46.3 for converting and unifying gene names^67^. We used SingleCellExperiment v1.12.0 for filtering and processing of feature counts matrix from scRNA-seq data^68^. We used edgeR v.3.32.1 and limma v3.46.0 for differential gene expression and gene set enrichment analysis^36,39^. We used zinbwave v1.12.0^30^ and Rtsne v0.15^69^ for dimension reduction, slingshot v1.8.0^34^ for trajectory analysis, and mclust v5.4.7^35^ for cluster analysis in the reduced dimensions. The plots were generated using ggplot v3.3.3^70^.

### Reporting summary

Further information on research design is available in the Nature Portfolio Reporting Summary linked to this article.

## Supplementary information

Supplementary Information

Description of Additional Supplementary Files

Supplementary Data 1

Supplementary Data 2

Supplementary Data 3

Reporting Summary

## Data Availability

The simulation data and results, processed bulk DNA-seq and scRNA-seq data for HGSOC and HER2+ data along with the results are available at [10.5281/zenodo.4950446]. The novel Smart-Seq3 sequencing data for HGSOC have been deposited in the European Genome-Phenome archive (EGA) under accession number EGAS00001006868. The DLP scDNA-seq data used for forming the pseudo-bulk data for HGSOC are available at the European Genome-Phenome archive with accession EGAS00001003190. The 10X single-cell RNA-seq data used for HGSOC are available at the European Genome-Phenome archive with accession EGAD00001004552. The novel Smart-Seq3 and whole-exome DNA sequencing HER2+ data are hosted on the federated EGA node in Sweden (EGA-SE) with accession number EGAS00001006851. The novel sequencing data have restricted access in line with the general data protection regulations (GDPR) of the European Union, which considers human sequencing data as sensitive personal information. The application for access will be granted if the subject of the applicants’ study where the data will be used is covered by the informed consent given by the individuals sequenced, and if there is ethical permission that covers the research project. Once the access is granted, the applicant may download and use the data as long as needed to complete the research. The remaining data are available within the Article, Supplementary Information or Source Data file. The source data are provided with this article. The GRCh37 of Human Genome release 75 is available for download from Ensembl [https://grch37.ensembl.org/info/data/ftp/index.html]. Source data are provided with this paper.

## Source data

Source Data

## Code availability

The PhylEx software is implemented in *C++* and its accompanying gene expression analysis code along with installation instructions and guides for conducting the analysis are available on Github at https://github.com/junseonghwan/PhylExAnalysisand version of the software used for analysis is available at https://zenodo.org/badge/latestdoi/335060186. The code for running other software is also provided in the above Github repository, with the settings used described in Supplementary Section 3.
