# Supplementary material for: Reconstructing clonal tree for phylo-phenotypic characterization of cancer using single-cell transcriptomics
Source: Nat Commun. 2023 Feb 22;14:982. doi: 10.1038/s41467-023-36202-y (PMC9946941; doi:10.1038/s41467-023-36202-y)
Supplement: Supplementary file 1 — Supplementary Information [file 41467_2023_36202_MOESM1_ESM.pdf]

Supplementary materials for Accurate reconstruction of clonal structure for phylo-phenotypic characterization of cancer clones using single-cell transcriptomics

Seong-Hwan Jun[1,2], Hosein Toosi[1], Jeff Mold[3], Camilla Engblom[3], Xinsong Chen[4], Ciara O'Flanagan[5], Michael Hagemann-Jensen[3], Rickard Sandberg[3], Samuel Aparicio[5,8], Johan Hartman[4,6], Andrew Roth*[5,7,8], and Jens Lagergren*[1]

[1]SciLifeLab, School of EECS, KTH Royal Institute of Technology, Stockholm, Sweden
[2]Current affiliation: Fred Hutchinson Cancer Research Center, Seattle, USA
[3]Department of Cell and Molecular Biology, Karolinska Institutet, Solna, Sweden
[4]Department of Oncology and Pathology, Karolinska Institutet, Solna, Sweden
[5]Department of Molecular Oncology, BC Cancer, Vancouver, BC, Canada
[6]Department of Clinical Pathology and Cytology, Karolinska University Laboratory, Stockholm, Sweden.
[7]Department of Computer Science, University of British Columbia, Vancouver, Canada
[8]Department of Pathology and Laboratory Medicine, University of British Columbia, Vancouver, Canada

Supplementary Note 1 - PhylEx probabilistic model

Marginalization over the copy number profiles for bulk data likelihood calculation

We expand on the description of the marginalization process given the major and minor copy numbers. Note that we do not know whether the variant allele is located on the major or the minor copy and that the number of variant copies is also unknown. Let A denote the reference allele and B denote the variant allele. For illustration, consider $(M_n, m_n) = (1, 1)$, i.e., copy number neutral. Then, the possible genotypes are A/B or B/A but the two are the indistinguishable and hence, $\mathcal{G}(1, 1) = \{A/B\}$. If $(M_n, m_n) = (2, 1)$, then $\mathcal{G}(M_n, m_n) = \{AA/B, BA/A, BB/A\}$. To see this, note that if the variant allele is on the major copy, then we have two possibilities, 1) both copies harbour the variant (BB/A) or 2) only one copy harbours the variant (BA/A). If the variant is on the minor copy and since the minor copy number is 1, the only possible case is AA/B. We use uniform prior over the possible elements of the genotype.

Estimating hyperparameters of scRNA-seq data

Our primary interest is in estimating α_n, β_n as a part of the preprocessing step. We describe a simple approach that we used in the data analysis. Our approach is to predict $\delta_{c,n}$ first, then estimate α_n, β_n using Beta-Binomial conjugacy. To predict $\delta_{c,n}$, we compute:

$$P(\delta_{c,n} = 1 | b_{c,n}, \alpha_n^0, \beta_n^0, \alpha_0, \beta_0, \delta_0) \propto P(b_{c,n} | \delta_{c,n} = 1, \alpha_n^0, \beta_n^0, \alpha_0, \beta_0,) P(\delta_{c,n} = 1 | \delta_0), \tag{1}$$

where α_n^0, β_n^0 quantify initial belief over the parameters for bi-allelic Beta distribution. We set $\alpha_n^0 = \beta_n^0 = 1$, which defines the Uniform distribution on $[0, 1]$, $\delta_0 = 0.5$, and $\alpha_0 = \beta_0 = 0.05$. Given $\delta_{c,n}$, the hyper parameter update is,

$$\alpha_n = \alpha_n^0 + \sum_{n:\delta_{c,n}=1} b_{c,n} \tag{2}$$

$$\beta_n = \beta_n^0 + \sum_{n:\delta_{c,n}=1} (d_{c,n} - b_{c,n}), \tag{3}$$

which is the standard update formula for Beta-Binomial hyperparameters.

Supplementary Note 2 - Simulated data generation

Simulating clone tree

We generate a binary tree with the root node representing the non-malignant ancestor. The root node has exactly one child, representing progenitor cancer clone with cellular prevalence of 0.5. We grow the tree starting from the progenitor clone such that each node has exactly two children; each child breaks half of the parent's remaining clone fraction: $\phi_u = 0.5\eta_{\rho(u)}$ where $\rho(u)$ denotes the parent of u. We terminate expansion of a lineage when the depth reaches the maximum specified or the cellular prevalence of the terminal node of the lineage falls below minimum threshold. In the simulated experiments, we set the maximum depth to 3 (depth of the root node is 0) and minimum cellular prevalence to 0.05. This results in a binary tree with eight nodes as shown in Supplementary Figure 8 **g**. We have an implementation that allows the cellular prevalence to be randomly sampled but generating the cellular prevalence in a determined manner creates for an interesting and challenging scenario. In particular, there are two pairs of clones that have the same cellular prevalence of 0.125 and 0.0625. Having clones with the same cellular fraction makes it difficult for reconstruction based on variant allele frequencies alone. To simulate the multifurcating tree, we again set the root node to be the healthy clone and it has exactly one child that represents the progenitor cancer clone. Starting from the progenitor cancer clone, we grow the tree by randomly selecting number of children from $\{1, 2, 3, 4\}$. The clone fraction of the children nodes are determined in the same way as for the binary tree. We stop the lineage expansion when the maximum depth of 3 or the minimum cellular prevalence of 0.05 is reached. Examples of trees generated for simulation studies is shown in Supplementary Figure 8 **h-i**.

Bulk data generation

To model cancer's complex structural variation and to study the effect of copy number misspecification, we use birth-death process to simulate copy number profiles. Birth-death process is parameterized by the birth rate and the death rate, with maximum copy number of 10 and the absorbing copy number of 0, i.e., once the copy number reaches 0, it does not evolve. We first construct two rate matrices, $\mathbb{Q}_0$ with minimum copy number state of 0 and $\mathbb{Q}_1$ with the minimum copy number state of 1 [3]. The transition matrices are obtained via matrix exponentiation, $\mathbb{P}_0 = \exp(\mathbb{Q}_0)$ and $\mathbb{P}_1 = \exp(\mathbb{Q}_1)$.

Let SNV n be assigned to node u of the tree. We initialize the copy number at the root node with the value of 2. We then separate copy number evolution into three parts. The first part is along the branches from the root node to u. We evolve the copy number using $\mathbb{P}_1$, this ensures that there is at least one copy by the time we get to node u. Let $X'_{n,u}$ be the copy number at node u, we then sample $Y'_{n,u} \sim \text{Binomial}(X'_{n,u} - 1, \xi)$, where $\xi \in (0, 1)$. Then, we set the variant copy number at u as $Y_{n,u} = Y'_{n,u} + 1$ and set the reference copy number as $X_{n,u} = X'_{n,u} - Y'_{n,u}$. The second part is copy number evolution starting at node u, which is initialized with copy number profile of $(X_{n,u}, Y_{n,u})$. We evolve $(X_{n,u}, Y_{n,u})$ independently using $\mathbb{P}_0$ to the leaf nodes. The third part is copy number evolution over all of the other branches, that is, the branches not in the path from root node to u and not in the subtree rooted at u. The copy numbers are evolved using $\mathbb{P}_0$ for these branches.

Once we have the full copy number profile at each clone, then we take the weighted averages,

$$\bar{X}_n = \sum_{v \in T} \eta_v X_{n,v} \tag{4}$$

$$\bar{Y}_n = \sum_{v \in T} \eta_v Y_{n,v} \tag{5}$$

$$\bar{D}_n = \sum_{v \in T} \eta_v (X_{n,v} + Y_{n,v}) \tag{6}$$

We round $\bar{X}_n, \bar{Y}_n$, then sort $(\bar{X}_n, \bar{Y}_n)$ to convert it to integer-valued major and minor copy numbers for PhylEx and other software used in the study. Note that these copy numbers provided as input to PhylEx does not fully capture the true copy number state of the cancer, which is as we desired to study the effect of scRNA-seq in mitigating the inaccuracies from the copy number detection step. The bulk data read counts

are generated as follows:

$$d_n \sim \text{Poisson}(d_0 \cdot \bar{D}_n/2) \quad (7)$$
$$b_n \sim \text{Binomial}(d_n, \xi_n), \quad (8)$$

where d_0 is the desired mean depth, set to $1,000$ in the simulation studies and $\xi_n = \bar{Y}_n/\bar{D}_n$, denoting the probability of observing a variant read. The division by factor of 2 arises when we consider $\bar{D}_n = 2$, e.g., when there is no copy number variation. In that case, to ensure the realized depth has mean d_0, we need to divide by 2. The birth rate of 1 and death rate of 0.2 was used in simulated data generation.

Simulating scRNA-seq data

Given the tree and the SNV-to-clone assignment, we first sample a cell-to-clone assignment for each of the cells. Assigning cell to a clone determines its genotype, call it $\mathcal{G}_c$. We randomly select a subset of SNVs to be expressed, denoted $\mathcal{E}_c$. For $n \in \mathcal{G}_c \bigcap \mathcal{E}_c$, we first sample the depth, $d_{c,n}$ from Poisson distribution with mean expression level e_0. Then, we sample from Bernoulli distribution to set $\delta_{c,n}$; the parameter of Bernoulli corresponds to bi-allelic expression probability – we used 0.2 in the simulation. If $\delta_{c,n} = 1$, we sample $b_{c,n}$ from Beta-Binomial$(d_{c,n}, \alpha_n, \beta_n)$. The hyperparameters α_n, β_n are sampled from uniform distribution with over $(0, max)$ with $max = 10$. If $\delta_{c,n} = 0$, we sample $b_{c,n}$ from Beta-Binomial$(d_{c,n}, \alpha_0, \beta_0)$ with parameters $\alpha_0 = \beta_0 = 0.01$. For $n \in \mathcal{E}_c \setminus \mathcal{G}_c$, we sample $b_{c,n}$ from Beta-Binomial$(d_{c,n}, \epsilon, 1-\epsilon)$, where $\epsilon = 0.01$ denotes the sequencing error (Supplementary Figure 7 **c**). As the loci n is expressed but the cell does not harbor the SNV, we expect to observe a variant read only in error. For $n \notin \mathcal{E}_c$, we set $d_{c,n} = b_{c,n} = 0$.

Supplementary Note 3 - Software settings

In this section, we describe the software settings for running PhylEx, TSSB/PhyloWGS, Canopy, B-SCITE, ddClone, and InferCNV. Each of the above methods except InferCNV either adopts a full Bayesian approach or offer a version that uses Bayesian sampling; we used 4 MCMC chains in parallel where applicable. PhylEx, TSSB/PhyloWGS, Canopy, B-SCITE, ddClone, and InferCNV were applied to simulated and HGSOC data. PhylEx, TSSB, and InferCNV were applied to HER2+ data as well as simulated and HGSOC data.

PhylEx and TSSB/PhyloWGS

PhylEx and PhyloWGS use TSSB prior, which is parameterized by $\lambda_0, \lambda, \gamma$. The support set for each of these parameters are bounded: $\lambda_0 \in [\lambda_0^{min}, \lambda_0^{max}]$, $\lambda \in [\lambda^{min}, \lambda^{max}]$, and $\gamma \in [\gamma^{min}, \gamma^{max}]$. Therefore, the hyperparameters to be specified are: $0 \leq \lambda_0^{min} \leq \lambda_0^{max} < \infty$; $0 \leq \lambda^{min} \leq \lambda^{max} < 1$; $0 \leq \gamma^{min} \leq \gamma^{max} < \infty$. The TSSB prior allows exploration of various tree topologies to best fit the data given these boundaries. In other words, it does not require *apriori* specification of the number of clones or whether the evolution is linear, bifurcating, or multifurcating. Our recommendation is to set the lower bounds to 0 and start with relatively large value for the max parameters: $\lambda_0^{max} = 10, \lambda^{max} = 0.8, \gamma^{max} = 0.5$. All three methods also require copy number information. PhyloWGS requires *subclonal* copy number information but we were not able to generate sensible copy number input even after following the software documentation. Therefore, we implemented a method underlying PhyloWGS based on our implementation of TSSB prior that takes in the same clonal copy number input as PhylEx – we simply refer to our implementation of PhyloWGS as TSSB. The copy number information is generated using TitanCNA for both PhylEx and TSSB.

All three methods share sequencing error probability, $0 < \epsilon \ll 1$. With advances in sequencing technologies, we recommend setting ϵ to a small value: $\epsilon \in \{0.001, 0.005, 0.01\}$. In addition, PhylEx requires hyper parameters α_0, β_0 for Beta-Binomial distribution for modelling mono-allelic expression for scRNA-seq data. From exploratory analysis of the real scRNA-seq data, we found that setting $\alpha_0 = \beta_0 = 0.01$ fit the data well.

Simulated data

For PhylEx and TSSB, we used $\lambda_0 = 10, \lambda = 0.8, \gamma = 0.5, \epsilon = 0.001, \alpha_0 = \beta_0 = 0.01$. We ran the slice sampler for 2,000 iterations, each iteration performed 2,000 Metropolis-Hastings iterations to sample the cellular

prevalences. For PhyloWGS, we used the default settings: $\lambda_0 = 25.0, \lambda = 0.25, \gamma = 1.0, \epsilon = 0.001$. The slice sampler was ran for 3,500 iterations with first 1,000 iterations as burn-in. Each iteration performed 5,000 Metropolis-Hastings iterations to sample the cellular prevalences.

HGSOC

We ran PhylEx and TSSB using $\lambda_0 = 2, \lambda = 0.5, \gamma = 0.5, \epsilon = 0.001, \alpha_0 = \beta_0 = 0.01$. We ran the slice sampler for 10,000 iterations, each iteration performed 2,000 Metropolis-Hastings iterations to sample the cellular prevalences. We ran PhylEx and TSSB with 20 chains to generate the standard error estimates on the performance measures, i.e., to ensure good performance is not achieved by chance.

HER2+

We ran PhylEx and TSSB using $\lambda_0 = 2, \lambda = 0.5, \gamma = 0.5, \epsilon = 0.01, \alpha_0 = \beta_0 = 0.01$. We ran the slice sampler for 30,000 iterations with 2,000 MH iterations to sample the cellular prevalences.

Canopy

Canopy requires specification of the number of clones, copy number information, and the minimum and maximum number of MCMC iterations to use. As the number of clones is typically unknown in practice, Canopy recommends to try a range of values and to select the number of clone yielding the highest likelihood. We specified values from 3 to 12 in all experiments where Canopy was applied. Canopy requires copy number information to be generated from Falcon copy number analysis software [4]. We used default settings to run Falcon. Finally, we used 4 chains and specified $10,000$ as the minimum MCMC iterations to use and $100,000$ as the maximum MCMC iterations.

B-SCITE

B-SCITE requires false positive and false negative parameters. The scRNA-seq data typically will have high false negative rate due to bursty expression whereas false positive rate may be low. In all experiments, we used the false positive rate of 0.01 and false negative rate of 0.2. We ran 4 replicates each with 20,000 MCMC iterations in all experiments.

ddClone

ddClone requires tumor content and copy number information to be specified. We use 1.0 as the tumor content in all of the experiments. Note that the tumor content is used by ddClone to parameterize the bulk data likelihood. Specifying the value of 1.0 as the tumor content simplifies the ddClone bulk data likelihood to rely on cellular prevalences of each clone in computing the bulk data likelihood, which is essentially the same approach as the one used by PhylEx. Hence, using the value of 1.0 allows to compare PhylEx to ddClone in the respective method's handling of the single cell data likelihood. Note also that HGSOC is a cancer cell-line data, which justifies specification of 1.0 as the tumor content. The copy number information is generated using TitanCNA as for PhylEx and TSSB. We ran 4 chains each with 20,000 MCMC iterations for both the simulated and HGSOC data.

Single cell genotyping for B-SCITE and ddClone

To run B-SCITE and ddClone on the HGSOC data, we needed to perform genotyping for each cell. We used BCF-tools version 1.10.2 'mpileup' followed by 'call' with option '-P 0.1 -mv'.

InferCNV

We have also used InferCNV to compare SNV clones to CNV clones. We used the denoising function and HMM with 6 states using 'subcluster' analysis mode. We used the default setting for all other parameters. InferCNV was applied to HGSOC and HER2+ data using the same settings.

References

[1] Ryan P Adams, Zoubin Ghahramani, and Michael I Jordan. Tree-structured stick breaking for hierarchical data. In *Advances in Neural Information Processing Systems*, pages 19–27, 2010.

[2] Wei Jiao, Shankar Vembu, Amit G Deshwar, Lincoln Stein, and Quaid Morris. Inferring clonal evolution of tumors from single nucleotide somatic mutations. *BMC bioinformatics*, 15(1):35, 2014.

[3] Geoffrey Grimmett and David Stirzaker. *Probability and random processes.* Oxford university press, 2020.

[4] Hao Chen, John M Bell, Nicolas A Zavala, Hanlee P Ji, and Nancy R Zhang. Allele-specific copy number profiling by next-generation dna sequencing. *Nucleic acids research*, 43(4):e23–e23, 2015.

Supplementary tables and figures

Supplementary Table 1: Full list of down regulated pathways in EF compared to ABCD clone (FDR < 0.01). The pathways related to the immune system are found to be down regulated. The first column is the name of the gene ontology. The second column is the p-value. The third column is the false discovery rate.

GeneOntology	P.Value	FDR
MHC protein complex	4.85e-15	1.32e-11
Antigen processing and presentation of endogenous antigen	6.40e-15	1.32e-11
MHC class I protein complex	6.57e-14	8.08e-11
Response to type I interferon	2.86e-11	1.60e-08
Antigen processing and presentation of endogenous peptide antigen	2.61e-10	1.14e-07
Positive regulation of T cell mediated cytotoxicity	7.96e-09	2.51e-06
Interferon gamma mediated signaling pathway	2.08e-08	5.72e-06
Regulation of T cell mediated cytotoxicity	2.37e-08	6.07e-06
Positive regulation of antigen processing and presentation	4.99e-07	9.59e-05
Detection of other organism	6.87e-07	0.00012799
Lumenal side of membrane	1.11e-06	0.00019995
Positive regulation of interleukin 1 beta production	1.28e-06	0.000224036
Positive regulation of interleukin 1 production	3.82e-06	0.000572277
Response to interferon gamma	6.86e-06	0.000937457
Peptide antigen binding	7.05e-06	0.000942833
Negative regulation of natural killer cell mediated immunity	1.08e-05	0.001205857
Negative regulation of natural killer cell mediated cytotoxicity	1.08e-05	0.001205857
Phagocytic vesicle	1.14e-05	0.001242962
Regulation of alpha beta T cell proliferation	1.15e-05	0.001242962
Regulation of T cell mediated immunity	1.53e-05	0.001563431
Positive regulation of type 2 immune response	1.59e-05	0.001597496
Negative regulation of cell killing	1.93e-05	0.001857438
Negative T cell selection	2.23e-05	0.002108729
Positive regulation of T cell mediated immunity	2.57e-05	0.002359283
Positive regulation of leukocyte mediated immunity	2.78e-05	0.00247885
Phagocytic vesicle membrane	5.80e-05	0.004752687
Positive regulation of production of molecular mediator of immune response	6.43e-05	0.005199606
Positive regulation of alpha beta T cell proliferation	6.85e-05	0.00526053
Positive regulation of lymphocyte mediated immunity	7.89e-05	0.005915636
Cellular response to interferon gamma	9.11e-05	0.006670378
Positive regulation of interleukin 12 production	9.48e-05	0.006857689
MHC class II protein complex	0.000131493	0.009186575

Supplementary Table 2: Summary statistics on genes used in the analysis of HER2+ breast cancer data. The first column lists the gene names. The second column indicates the number of cells with coverage on the gene. The third column is the mean number of reads over the cells at the gene. Source data are provided as a Source Data file.

Gene	Cell Count	Mean Reads
PIK3R3	310	66.74
FN1	106	19.36
WNT10A	0	0
CACNA2D2	166	3.92
DKK2	0	0
PITX2	0	0
MDC1	108	4.50
EZH2	241	18.25
COL4A5	156	7.81
PRKDC	307	34.13
PRKACG	0	0
IL2RA	0	0
MAP3K8	306	122.36
DDX50	111	4.73
ACVR1B	219	9.59
FGF14	4	0.22
POLE2	47	1.43
FOS	377	3003.54
VPS33B	102	4.50
TP53	271	15.35
NF1	331	123.16
CDC6	70	5.37
CACNG4	241	19.44
ETS2	223	27.08

Supplementary Table 3: Performance metric of PhylEx on HGSOC when hyperparameter supports are varied. The first column lists the hyperparameters. The second, third, and fourth columns are clustering metrics used for comparison, where higher value is preferred. The last column is the ancestral reconstruction error metric, where lower value is preferred. The standard error estimates were obtained by repeating the experiments 20 times. Source data are provided as a Source Data file.

$(\lambda_0^{max}, \lambda^{max}, \gamma^{max})$	V-Measure	Adj. Rand Index	Adj. Mut Info	Anc. Recon Err
$(1, 0.2, 0.5)$	0.859 ± 0.0590	0.851 ± 0.1043	0.827 ± 0.07184	0.0498 ± 0.0348
$(5, 1, 0.2)$	0.868 ± 0.0188	0.884 ± 0.0227	0.836 ± 0.0252	0.0394 ± 0.0099
$(5, 1, 1)$	0.870 ± 0.0132	0.888 ± 0.0164	0.839 ± 0.0175	0.0379 ± 0.0077
$(10, 1, 1)$	0.863 ± 0.0217	0.882 ± 0.0180	0.830 ± 0.0276	0.0378 ± 0.0046

Supplementary Table 4: Table of notation used for random variables, their descriptions, and whether they are inferred, marginalized, pre-estimated, or taken as user input.

T	Clonal tree	Inferred
$\boldsymbol{z}$	SNV assignment	Inferred
$\boldsymbol{\phi}$	Cellular prevalence	Inferred
$\lambda_0, \lambda, \gamma$	TSSB hyperparameters	Inferred
ζ_c	Cell assignment to clone	Inferred/Marginalized
$\delta_{c,n}$	Indicator of bi-allelic expression for cell at loci	Marginalized
δ_n^0	Prior probability of bi-allelic expression	User input
ϵ	Sequencing error probability	User input
α_0, β_0	Mono-allelic expression hyperparameters	User input
α_n, β_n	Bi-allelic expression hyperparameters for loci	Pre-estimated
b_n, d_n	Variant read counts and total read counts for loci	Observed
$b_{c,n}, d_{c,n}$	Variant read counts and total read counts for cell at loci	Observed
M_n, m_n	Major and minor copy number profiles for loci	Pre-estimated

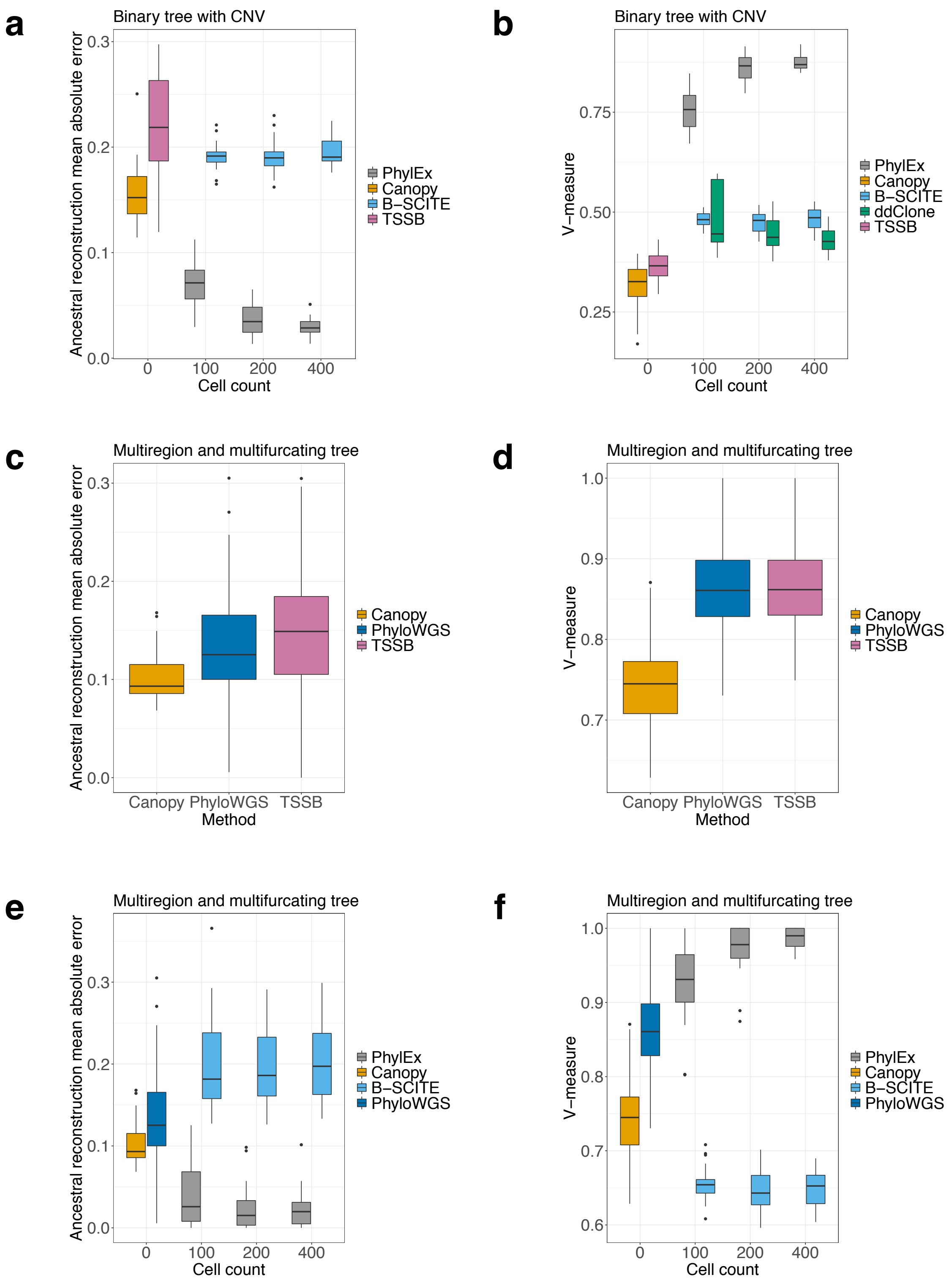

Supplementary Figure 1: Supplementary simulation analysis results. Data generated with 100 SNVs with 20 replicates to derive the error bars. a-b. Binary tree with copy number evolution on mean absolute reconstruction error and V-measure clustering metric. c-d. Comparison of bulk based deconvolution methods on binary tree. e-f. Comparison of bulk based deconvolution methods on multifurcating tree using multi-region bulk data. The box plot shows the median and inter-quantile range (IQR) at the 1st and the 3rd quantiles; the top (bottom) whisker indicates the maximal (minimal) point no further than 1.5 $\times$ IQR from the third (first) quantile. Source data are provided as a Source Data file.

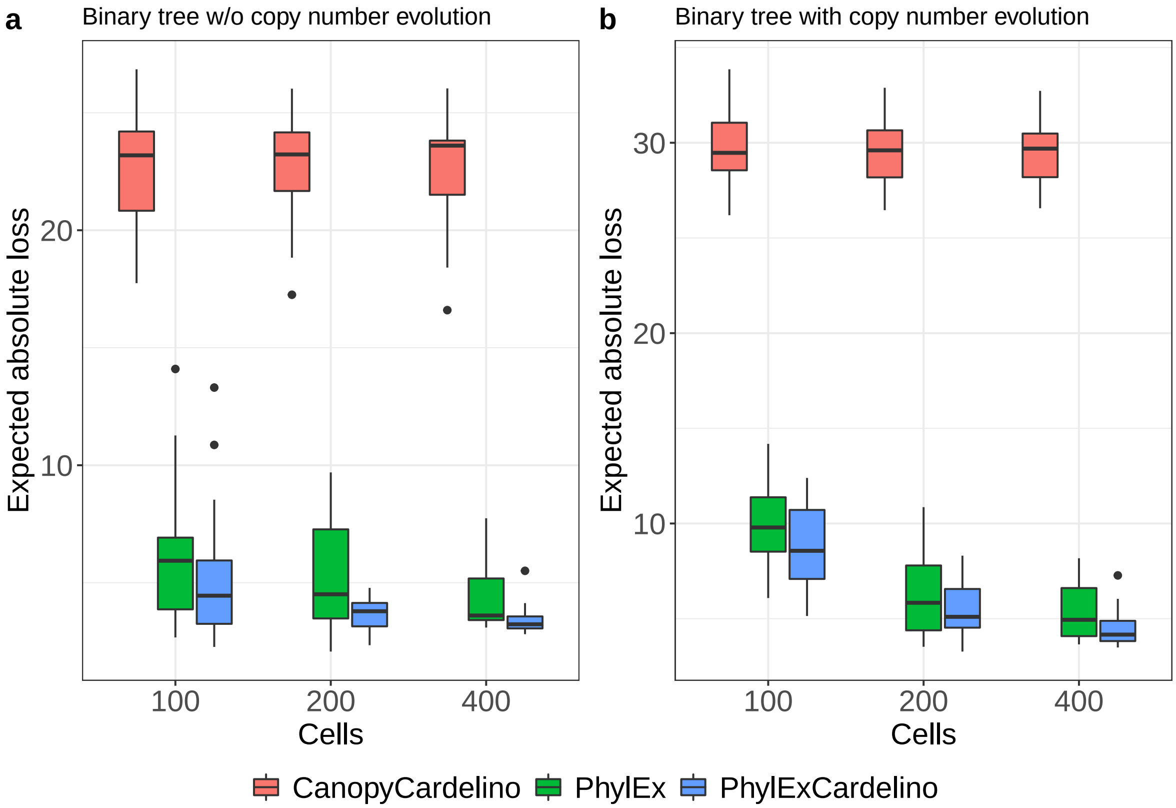

Supplementary Figure 2: Comparison of mapping of scRNA-seq to clones using two stage approach (Canopy-Cardelino) to clones inferred using PhylEx (PhylExCardelino and PhylEx) on expected loss. Data simulated from binary trees a. without copy number variants and b. with copy number evolution. Data generated with 100 SNVs and simulation repeated over 20 replicates to derive the error bars. The box plot shows the median and inter-quantile range (IQR) at the 1st and the 3rd quantiles; the top (bottom) whisker indicates the maximal (minimal) point no further than $1.5 \times$ IQR from the third (first) quantile. Source data are provided as a Source Data file.

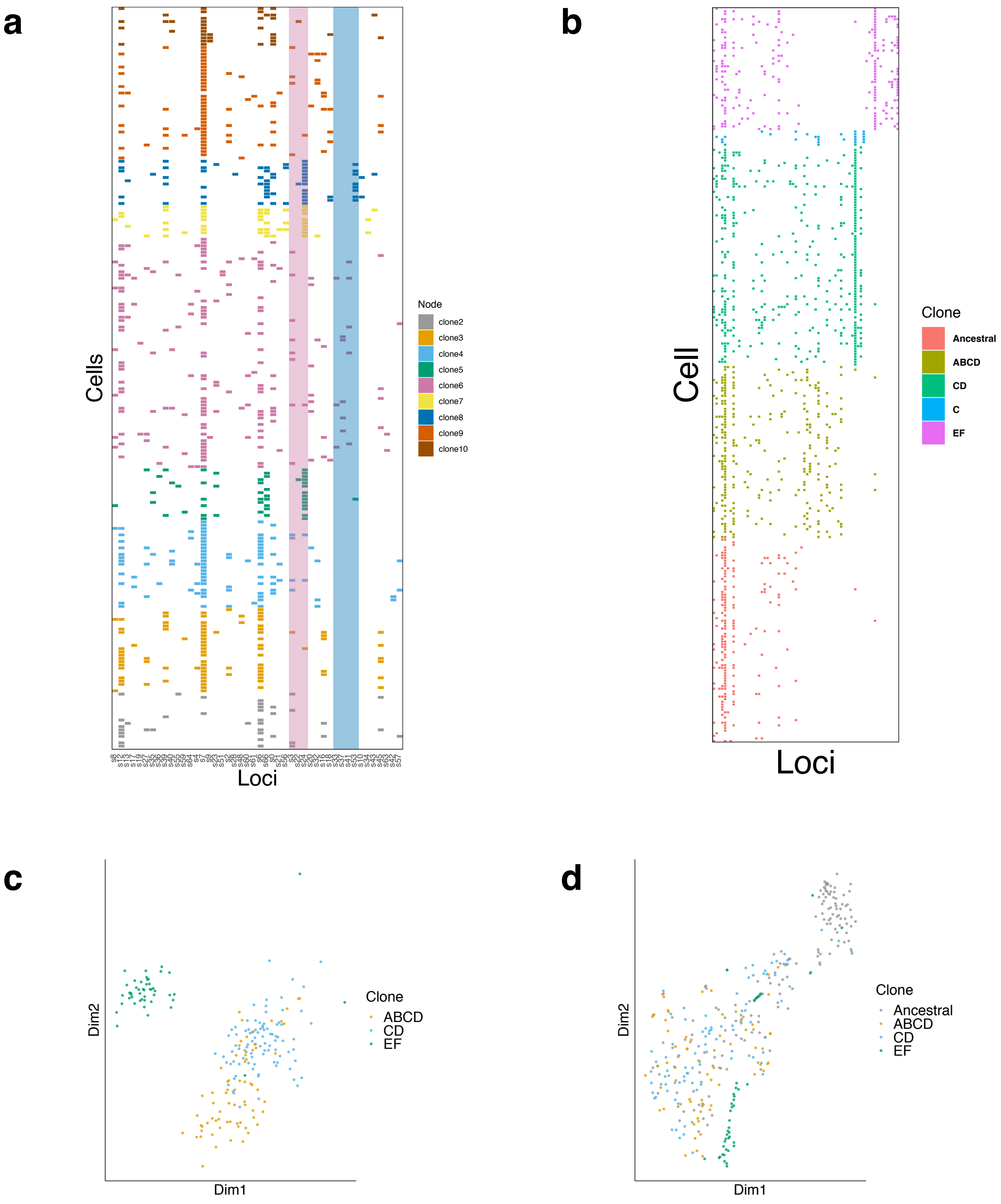

Supplementary Figure 3: Additional HGSOC cell line biological analysis results. Plot of variant read counts as absence/presence heatmap after co-clustering by cells and SNVs. a. using Cardelino on Canopy tree. The mutations exclusive to clones 6 is annotated by red and similarly for clone 8 in blue. Cells assigned to other clones are seen to carry evidence of mutation on these loci, demonstrating the problem of two-step approach. b. using PhylEx. c. Plot of gene expressions of cells after performing ZINB-WaVE dimension reduction – plotted without ancestral cells. d. Plot of gene expressions of cells after performing t-SNE dimensions reduction. Source data are provided as a Source Data file.

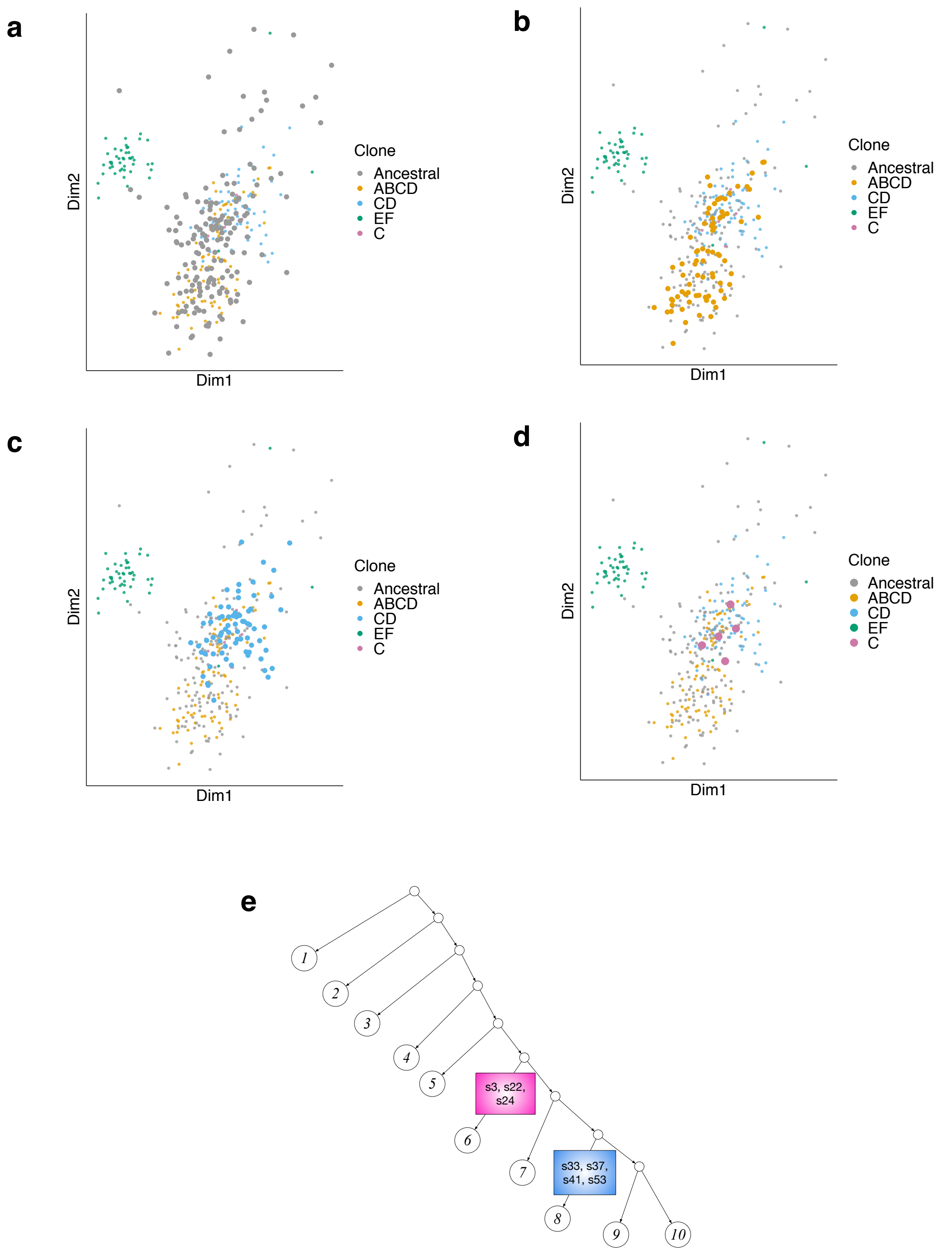

Supplementary Figure 4: Additional HGSOC cell line biological analysis results. Plot of gene expressions of cells after performing ZINB-WaVE dimension reduction with dot sizes expanded for a. clone ancestral, b. clone ABCD, c. clone CD, d. clone C. e. Tree inferred by running Canopy on HGSOC data. Mutations occuring exclusively for Clone 6 (pink) and Clone 8 (blue) are highlighted. Source data are provided as a Source Data file.

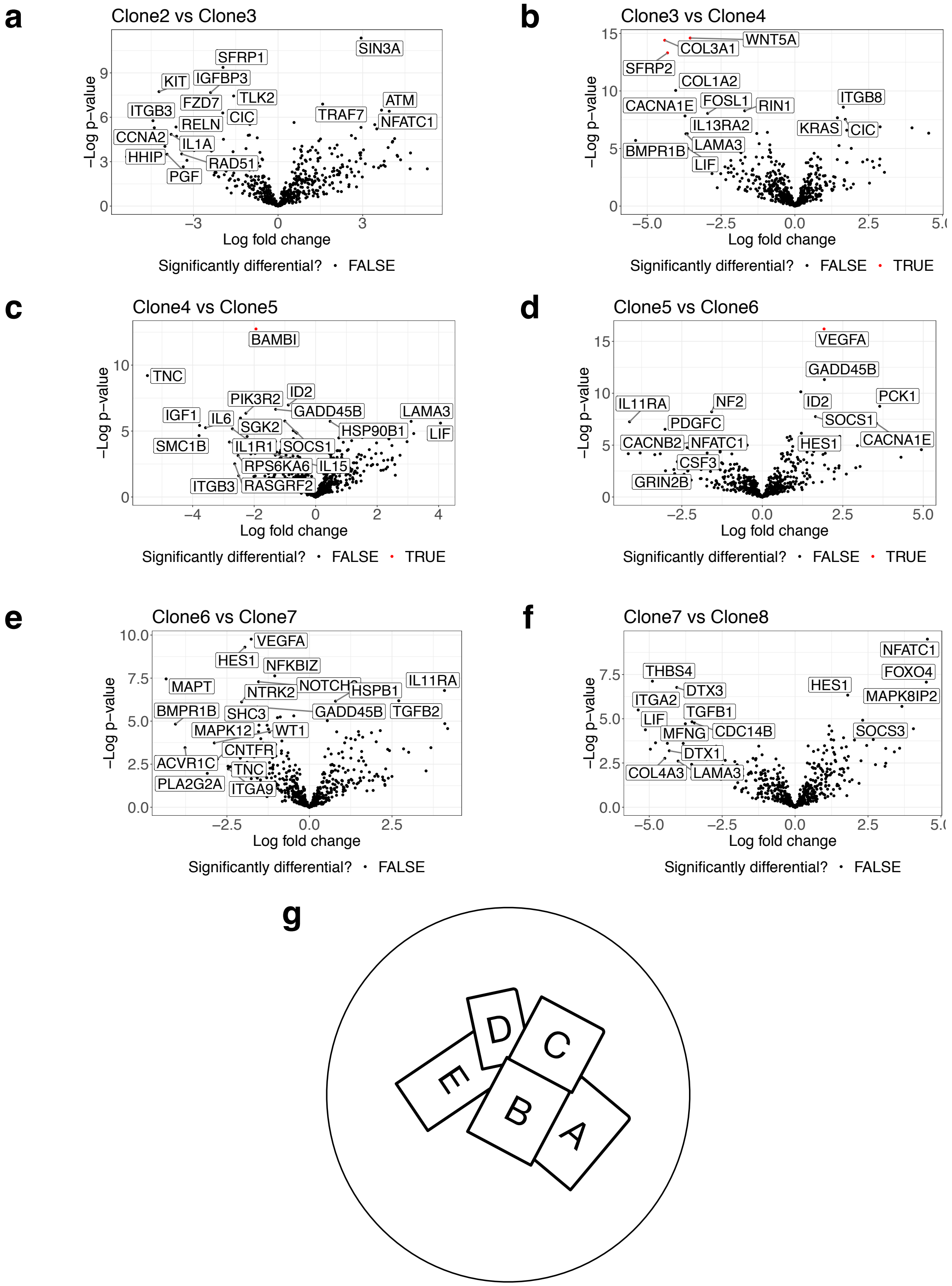

Supplementary Figure 5: a-f. Volcano plots comparing all parent-child clone pairs. g. Schematic depiction of the locations of multi-regional sampling for HER2+. The proximity of the regions A, B, and C explains the homogeneity in their clone fractions. The region D is remote from region A and the region E does not have any overlap with regions A and C, which explains the differences in the clone fractions in regions D, E to regions A, B, and C. Source data for a-f are provided as a Source Data file.

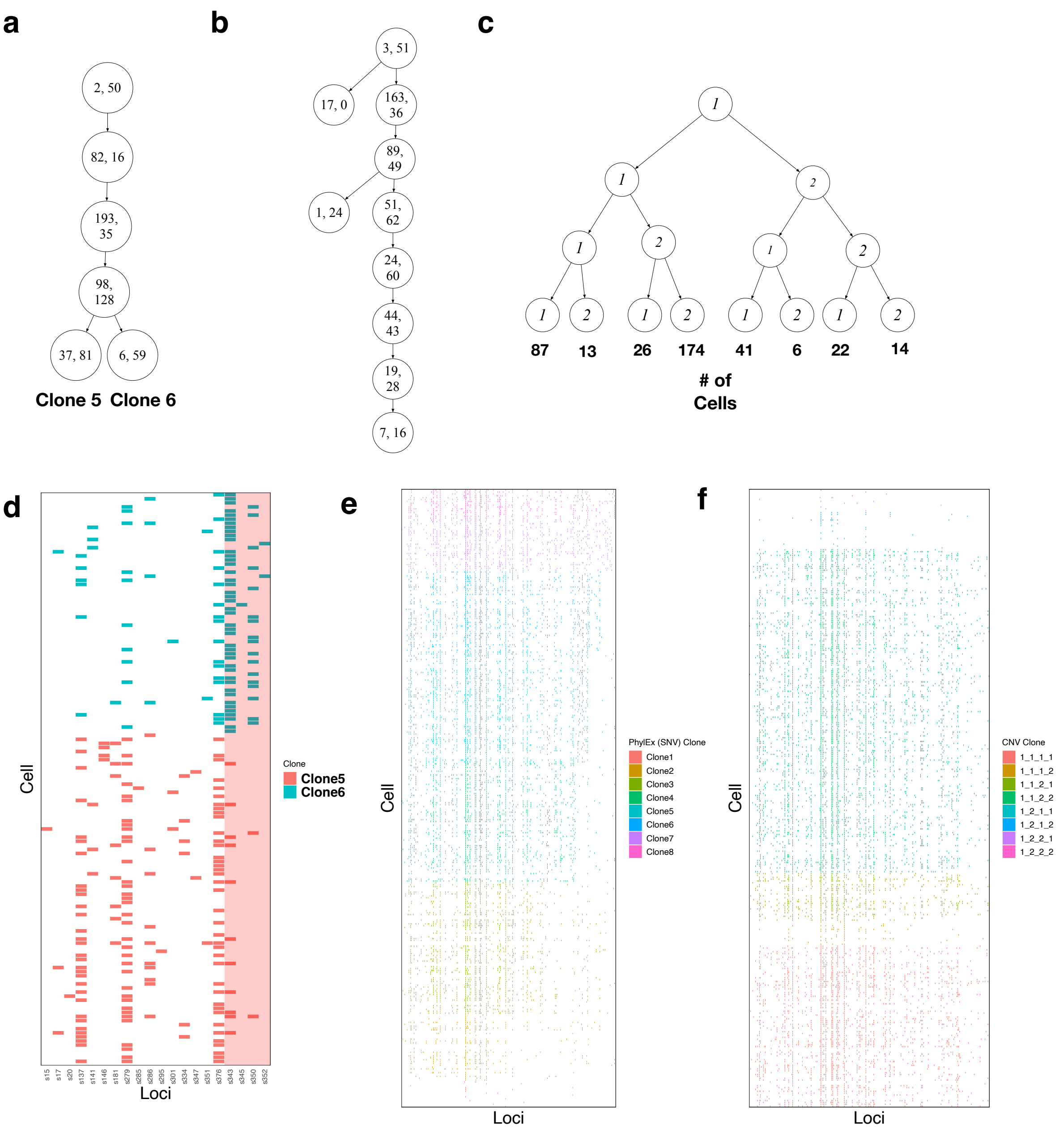

Supplementary Figure 6: Additional HER2+ breast cancer analysis results. a. tree inferred from TSSB, b. tree inferred from PhylEx before pruning. The number inside the node indicates the # of SNVs and the # of cells assigned to each clone. c. CNV clones (leaf nodes) and the tree inferred by InferCNV. The number below the leaf node indicates the # of cells assigned to each clone. d. Plot of binarized variant read counts to indicate absence/presence of variant read for each loci for cells on clone 5 and clone 6 from TSSB tree shown in a. e. mutation and cells ordered by PhylEx clones supporting linear expansion. f. cells ordered by CNV clones inferred from InferCNV tree shown in c. Source data are provided as a Source Data file.

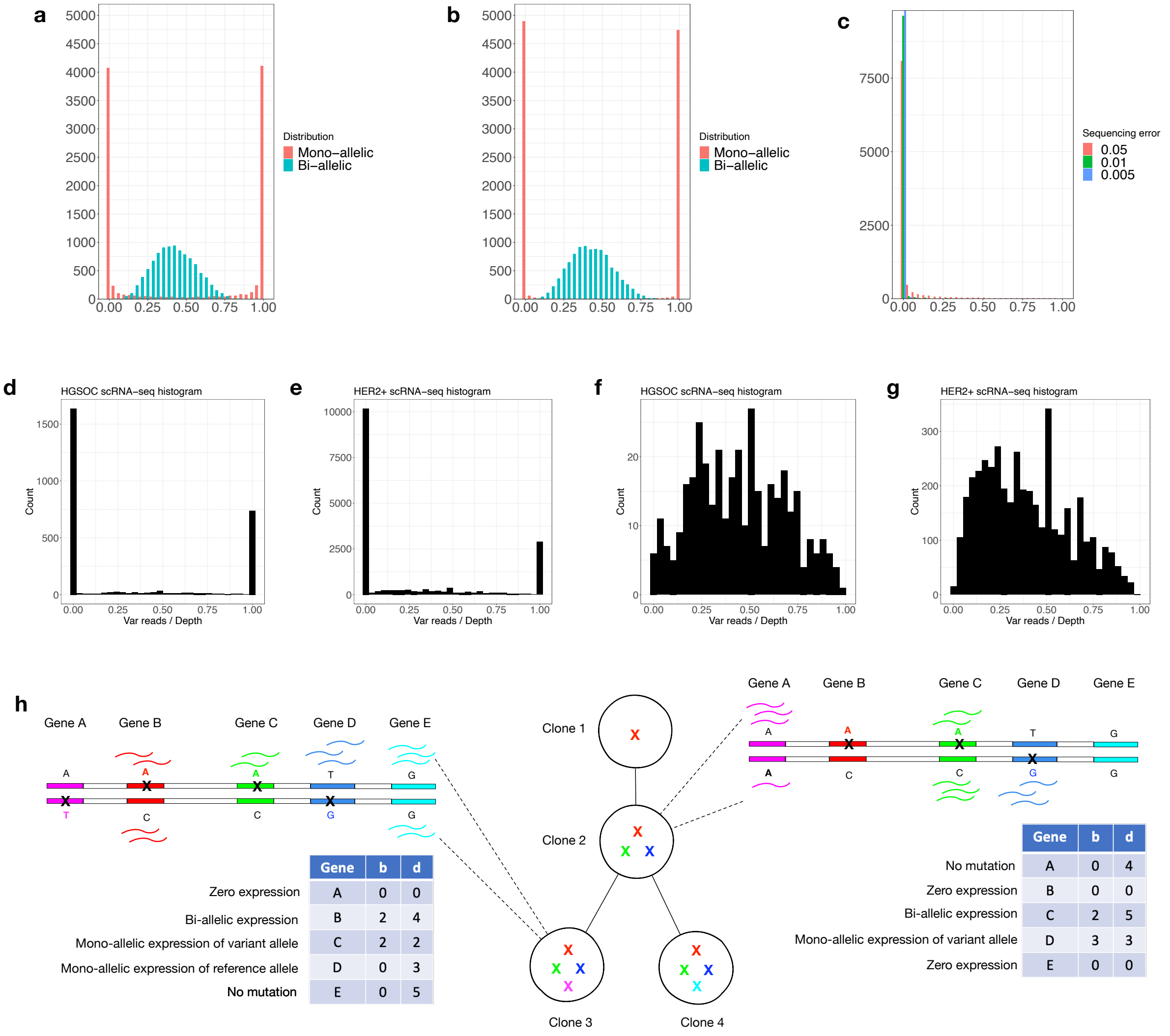

Supplementary Figure 7: a. Beta-Binomial mixture distribution with $\alpha_0 = \beta_0 = 0.05$, b. $\alpha_0 = \beta_0 = 0.01$; the values for the biallelic hyper parameters supplied are $\alpha_n = 5, \beta_n = 7$. As α_0, β_0 decreases, the mass in the mono-allelic distribution concentrates near 0 and 1. c. The ratio of variant reads to depth for the error distribution for $\epsilon \in \{0.05, 0.01, 0.005\}$. Plot of scRNA-seq reads for HGSOC and HER2+ data. d,e. Plot of the ratio of variant reads to depth for scRNA-seq across all loci. f-g. Plot of the ratio of variant reads to depth for scRNA-seq for subset of the data without the extremes at 0 and 1. h. Gene expression profile of a cell taken from Clone 3: Gene A is not expressed; both alleles of Gene B are expressed; Genes C and D are mono-allelic expression of variant and reference alleles; Gene E is not mutated and hence, only the reference allele can be expressed. We use b and d to denote variant read counts and read depth. Source data for a-g are provided as a Source Data file.

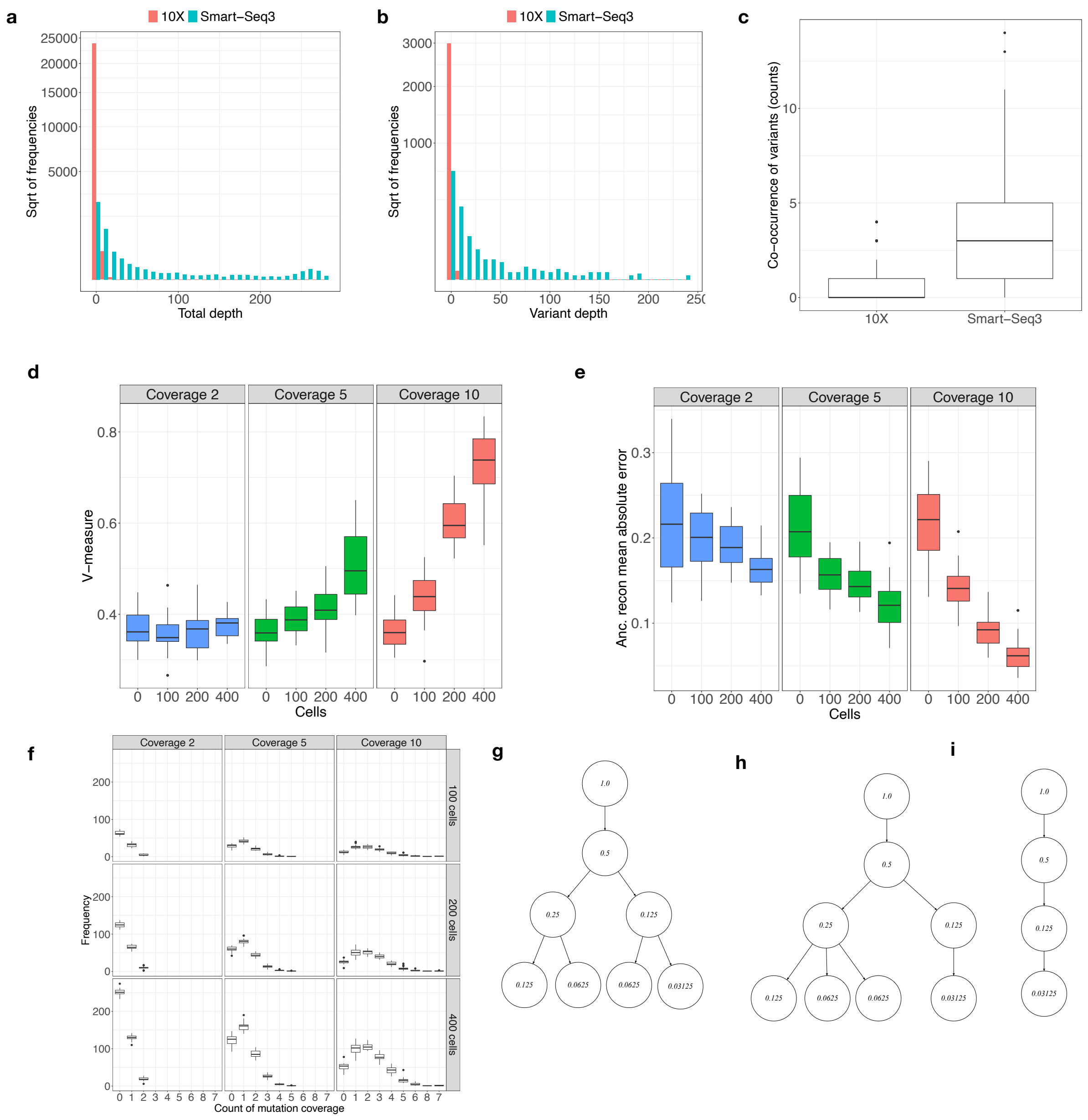

Supplementary Figure 8: a-b. Histogram of total depth and variant depth across all loci over all cells sequenced from HGSOC cell-line using 10X and Smart-Seq3. c. The boxplot showing the number of mutations co-occurring in cells. d-e. V-measure metric and ancestral reconstruction error to demonstrate the effect of coverage on the performance of PhylEx. f. Number of mutations covered by cells in the simulated dataset at different coverage rates (columns) and cells (rows). The boxplot is generated with 20 replicates for each experimental condition and shows the median and inter-quantile range (IQR) at the 1st and the 3rd quantiles. The top (bottom) whisker indicates the maximal (minimal) point no further than $1.5 \times$ IQR from the third (first) quantile. g. Binary tree used in the simulation. h. An example of a multifurcating tree used in the simulation. i. A linear tree used in the simulation. Source data for a-f are provided as a Source Data file.

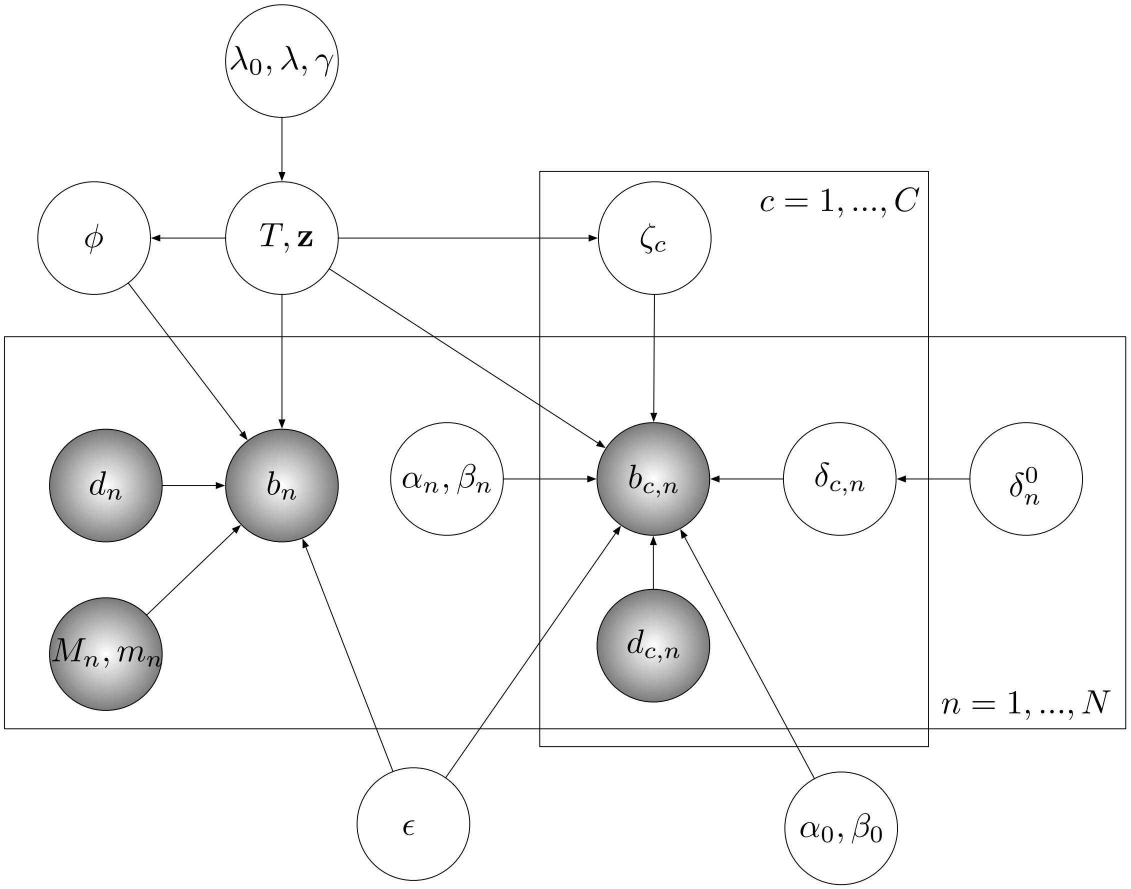

Supplementary Figure 9: PhylEx probabilistic model depicted as graphical model.
