# Supplementary material for: Reconstructing clonal tree for phylo-phenotypic characterization of cancer using single-cell transcriptomics
Source: Nat Commun. 2023 Feb 22;14:982. doi: 10.1038/s41467-023-36202-y (PMC9946941; doi:10.1038/s41467-023-36202-y)
Supplement: Supplementary file 2 — Description of Additional Supplementary Files [file 41467_2023_36202_MOESM2_ESM.docx]

Description of Supplementary Data

Supplementary Data 1: List of SNV used in the analysis of HGSOC. The first column is the SNV ID, the second and third columns are chromosome and genomic position, the fourth column contains the annotation from the study where the data originates from, and the last column indicates SNVs excluded from evaluation due to inconsistent annotation from the original study.

Supplementary Data 2: List of oligonucleotides and their information.

Supplementary Data 3: List of antibodies and their information.
